# Evolutionary trajectories and zoonotic potential of a PB2 mutation triad (I147T, K339T, and A588T) in avian influenza viruses

**DOI:** 10.1186/s13567-025-01680-z

**Published:** 2025-12-08

**Authors:** Seung-Eun Son, Se-Hee An, Chung-Young Lee, Jin-Ha Song, Ho-Won Kim, Seung-Ji Kim, Seung-Min Hong, Hyuk-Joon Kwon, Kang-Seuk Choi

**Affiliations:** 1 Laboratory of Avian Diseases, College of Veterinary Medicine, Seoul National University, Seoul, 08826 Republic of Korea; 2 Avian Influenza Research & Diagnostic Division, Animal and Plant Quarantine Agency, Gimcheon, 39660 Republic of Korea; 3 Department of Microbiology, School of Medicine, Kyungpook National University, Daegu, 41944 Republic of Korea; 4 Research Institute for Veterinary Science, College of Veterinary Medicine, Seoul National University, Seoul, 08826 Republic of Korea; 5 Farm Animal Clinical Training and Research Center (FACTRC), Institutes of Green Bio Science and Technology (GBST), Seoul National University, Pyeongchang, 25354 Republic of Korea; 6 Laboratory of Poultry Medicine, Department of Farm Animal Medicine, College of Veterinary Medicine and BK21 PLUS for Veterinary Science, Seoul National University, Seoul, 08826 Republic of Korea; 7 GeNiner Inc., Seoul, 08826 Republic of Korea

**Keywords:** Avian influenza virus, zoonosis, polymerase basic protein 2, host adaptation, highly pathogenic avian influenza virus, poultry vaccination

## Abstract

Efficient replication of influenza A viruses (IAVs) requires balanced activities of hemagglutinin (HA), neuraminidase (NA), and the RNA polymerase complex, whose functions are strongly influenced by PB2 mutations. We previously revealed three distinct evolutionary pathways for PB2 mutations, with two pathways leading to the emergence of viral strains responsible for human seasonal infections and the 2009 pandemic, and a third pathway giving rise to H5Nx highly pathogenic avian influenza viruses (HPAIVs) defined by a triad of mutations (I147T, K339T, and A588T) that occasionally spill over to humans. Here, we investigated the zoonotic risk posed by this triad and elucidated its evolutionary relationship with HA, NA, and vaccination. Recombinant PR8 and clade 2.3.2.1c H5N1 viruses carrying the triad replicated efficiently in embryonated chicken eggs and had moderate replication efficiency in mammalian cells; moreover, mice infected with these viral strains exhibited milder weight loss and lower lung titers than those infected with the E627K-carrying strain. Sequence analysis of H5Nx viruses revealed early emergence and long-term persistence of the triad across diverse genotypes, which was closely linked to HA glycosylation and NA-stalk truncation. However, the prevalence of these viral strains declined significantly after successive H5 poultry-vaccination campaigns. These data indicate that the triad provides a replication advantage compatible with both poultry and mammalian hosts but confers only moderate mammalian pathogenicity and that sustained vaccination can restrain the spread of viral strains with these mutations. Continuous molecular surveillance of PB2 alongside HA and NA remains essential for preventing H5Nx zoonotic threats.Please check and confirm that the authors and their respective affiliations have been correctly identified and amend if necessary.I have checked and modifed the email addresses of the corresponding authors. I confirm the information of other authors are correct.

**Supplementary Information:**

The online version contains supplementary material available at 10.1186/s13567-025-01680-z.

## Introduction

Avian influenza viruses (AIVs) are enveloped, segmented, negative-sense RNA viruses that evolve rapidly through mutation and reassortment, enabling cross-species transmission [1, 2]. Wild waterfowl serve as natural reservoirs, while dense populations of domestic poultry promote the emergence of replication-competent strains with zoonotic potential, posing significant public health concerns [3–5].

Following host cell entry, AIVs exploit the host machinery for genome release, nuclear import, transcription, and replication [5]. The viral polymerase subunit PB2 cap-snatches host messenger RNA (mRNA) to initiate transcription, and its amino acid changes often determine host range [5–7]. The E627K mutation, for example, increases polymerase activity in humans by interacting with the host factor ANP32B and suppressing RIG-1 signaling; however, it can reduce fitness in chickens [8–10].

We previously characterized a prototypic PB2 gene from the H9N2 virus A/chicken/Korea/01310/2001 that lacks canonical mammalian adaptation markers and attenuates highly pathogenic strains in mice [11]. The addition of three permissive substitutions (I66M, I109V, and I133V; collectively MVV) restored replication and acted as a prerequisite for the acquisition of additional mammalian adaptive mutations [12]. We further defined a stepwise evolutionary pathway by which PB2 adapts to mammalian hosts, contributing to the emergence of the viral strains responsible for seasonal influenza and the 2009 H1N1 pandemic and H5Nx highly pathogenic avian influenza viruses (HPAIVs) [13]. In particular, the accumulation of three additional mutations (I147T, K339T, and A588T) alongside MVV (MVV/I147T/K339T/A588T; hereafter MVVTTT_588_) has become more prevalent in H5Nx viruses, raising concerns about their mammalian pathogenic potential [13, 14]. Another arrangement in which I147T, K339T, and E627K (MVV/I147T/K339T/E627K; hereafter MVVTTK_627_) are present alongside MVV also appeared repeatedly in avian H5Nx isolates, despite potentially reducing fitness in chickens. The presence of E627K highlights the need to elucidate the host environment that gives rise to such high-risk mutations.

Influenza virus evolution is fundamentally multigenic, and coordinated changes in surface glycoproteins are well established. Hemagglutinin (HA) and neuraminidase (NA) coevolve through receptor-binding mutations, including alterations in the secondary sialic acid-binding site and NA-stalk truncations, which are events repeatedly observed during transmission from wild birds to poultry or serial egg passage [15–22]. Internal-gene crosstalk, such as NA-PB1 packaging compatibility, further underscores this interdependence [23]. However, whether PB2 follows a similar coadaptive trajectory with HA and NA remains unknown.

In addition to such intrinsic segment compatibilities, AIV evolution is strongly shaped by extrinsic immune pressures. Among these, host- and vaccine-induced antibodies exert the most consistent selective force on HA and NA. Successive H5 vaccines have driven immune-escape mutations adjacent to the HA receptor-binding site and mutations that give rise to N-linked glycosylation sites (NGSs), exemplified by 129-NGS in H5N6 and 144-NGS in H5N1 [24, 25]. Our previous work revealed that such vaccine pressure reshaped glycosylation patterns in clade 2.3.2.1c H5N1 viruses [26]. Building on these observations, we focus here on the presence of HA NGSs (129-, 144-, and 158-NGS) and NA-stalk length as sentinel markers to analyze whether the PB2 mutation triad MVVTTT_588_ coevolves with surface-gene adaptations under field vaccination programs. This integrated framework provides a rationale to evaluate the HA–NA–PB2 nexus in contemporary H5Nx HPAIVs and to determine how such interactions influence zoonotic risk.

In this study, we investigated the zoonotic risk and interdependence of the MVVTTT_588_ triad. Using reverse genetics, we generated recombinant PR8 (H1N1) and clade 2.3.2.1c H5N1 strains by introducing I147T, K339T, and A588T in a stepwise manner. The effects of these mutations on polymerase activity and viral replication efficiency were evaluated in 293T cells, embryonated chicken eggs (ECEs), mammalian cell lines, and mice. Complementary sequence analyses of the PB2, HA, and NA genes traced the global emergence of MVVTTT_588_ and E627K across various hosts, integrating HA and NA genotypes with regional vaccination timelines. By linking laboratory phenotypes with evolutionary patterns, we identified the drivers and risks associated with this PB2 adaptation pathway.

## Materials and methods

### Cells, eggs, and viruses

Madin‒Darby canine kidney (MDCK) and 293T cells were acquired from the Korean Collection for Type Cultures (KCTC, Dajeon, Republic of Korea), and Calu-3 cells were purchased from the Korean Cell Line Bank (KCLB, Seoul, Republic of Korea). MDCK and 293T cells were maintained in Dulbecco’s modified Eagle’s medium (DMEM) supplemented with 10% fetal bovine serum (FBS; Life Technologies Co., Carlsbad, CA, USA), and Calu-3 cells were maintained in minimum essential medium (MEM)/F12 supplemented with 10% FBS (Life Technologies, Carlsbad, CA, USA). Specific pathogen-free (SPF) eggs were purchased from VALO Biomedia (Osterholz-Scharmbeck, Lower Saxony, Germany) and incubated at 37 °C for 10 days and used for the experiments. Also, 293T cells were used exclusively for reverse-genetics rescue and mini-genome assays, whereas replication-kinetics experiments were conducted in MDCK and Calu-3 cells.

The A/Puerto Rico/8/1934 (H1N1) (PR8) and PR8-derived recombinant viruses containing mutated PB2 genes derived from the A/chicken/Korea/01310/2001 (H9N2) (01310) virus were generated. In addition, PR8-derived recombinant clade 2.3.2.1c H5N1 viruses with mutated PB2 genes were generated using the HA and NA genes derived from the A/Mandarin duck/Korea/K10-483/2010 (H5N1) (K10) virus. The viruses were generated using Hoffmann’s eight-plasmid DNA transfection system, as previously described [27]. Briefly, seven protein-coding genome segments of the PR8 virus (HA, NA, PB1, PA, NP, M, and NS) and two segments of the K10 virus (HA and NA) were cloned and inserted into a bidirectional pHW2000 vector, and the 01310 PB2 sequence was mutated to contain the mutations. One day before transfection, 293T cells were seeded in six-well plates, and a mixture containing equal amounts of each plasmid was transfected using Plus reagents and Lipofectamine 2000 (Life Technologies) according to the manufacturer’s protocol. One milliliter of Opti-MEM (Life Technologies) and a 4 µg/well concentration of l-1-tosylamido-2-phenylethyl chloromethyl ketone (TPCK)-treated trypsin (Sigma-Aldrich, St. Louis, MO, USA) were added to the wells after 24 h. The supernatants were harvested 1 day later, and 0.2 mL of supernatant was used to inoculate 10-day-old SPF embryonated chicken eggs (ECEs), which were subsequently incubated for 3 days.

The generation of the recombinant viruses was confirmed via a hemagglutination assay in which 1% chicken red blood cells (RBCs) were used, and the sequences of the mutant viruses were confirmed via polymerase chain reaction (PCR). The viral titers were measured after the eggs were passaged at least two times. Harvested viruses were serially diluted from 10^–1^ to 10^–9^ in tenfold increments, and each dilution was injected into four 10-day-old SPF ECEs or MDCK cells seeded in a 96-well plate. After incubation for 3 days at 37 °C, the 50% chicken embryo infectious dose (EID_50_) and 50% tissue culture infectious dose (TCID_50_) were calculated via the Spearman–Karber method [28].

### Generation of mutated 01310 PB2 plasmids and evaluation of relative luciferase activity via a mini-genome assay

The PB2 genes of 01310 (H9N2) and the HA and NA genes of K10 (H5N1), which were previously cloned and inserted into Hoffmann’s bidirectional vector pHW2000, were used in this study [12, 13, 29]. Site-directed mutagenesis was performed to introduce MVV, I147T, K339T, A588T, and E627K mutations into the plasmid using a Muta-Direct Site-Directed Mutagenesis Kit following the manufacturer’s protocol (iNtRON Biotechnology, Sungnam, Republic of Korea). The inserted sequences were confirmed using the primers CMV-F and bGH-R.

To evaluate the effect of mutated PB2 genes on polymerase activity, we used the pHW-NP-Luc plasmid as previously described [12]. Briefly, 293T cells in 12-well cell culture plates were cotransfected with pHW-NP-Luc (0.1 µg per well) and the 01310 PB2, PR8 PB1, PA, and NP genes. Additionally, 0.1 µg of the *Renilla* luciferase plasmid pRL-TK (Promega, Madison, WI, USA) was cotransfected to serve as an internal control to normalize variations in transfection efficiency. Twenty-four hours after transfection, the cells were harvested, and luminescence was measured using the Dual-Glo Luciferase Assay System (Promega) following the manufacturer’s instructions with a TECAN Infinite 200 Pro machine (Tecan Benelux bv, Giessen, Netherlands). The luciferase activity was compared with and normalized to the polymerase activity of PR8 PB2 or 01310 PB2. Additionally, PB2 variants carrying mutations prevalent among human seasonal flu (MVV-9N-199S-271A-526R-588T-590S-627K-674T-702R; HIB-9-2) and A(H1N1)pdm09 virus (MVV-147T-271A-588T-590S-591R; SIB-5-1) were also compared using previously mutated 01310 PB2 plasmids [13]. All experiments were performed in triplicate, and the results are shown as averages.

### Viral growth kinetics in mammalian cells

To evaluate the replication ability of the mutant viruses in mammalian cell lines, MDCK and Calu-3 cells were seeded in 12-well plates (5 × 10^5^ cells/mL). After 24 h, the confluent cells were washed twice with phosphate-buffered saline (PBS), and a 0.001 multiplicity of infection (MOI) of virus diluted in DMEM [supplemented with 1% bovine serum albumin (BSA) (Fraction V) (MP Biomedicals), 20 mM HEPES, penicillin‒streptomycin (Thermo Fisher Scientific), and 1 µg/mL TPCK-treated trypsin (Sigma-Aldrich)] was inoculated into MDCK and Calu-3 cells. After 1 h of incubation, the inoculated virus was removed via two washes with PBS, and 1 mL of fresh medium was added and incubated for 3 days. The cell supernatants were harvested at 0, 24, 48, and 72 h post-inoculation, and the viral titer at each time point was measured using the 50% tissue culture infective dose (TCID_50_). MDCK cells prepared in 96-well plates were inoculated with tenfold dilutions of cell supernatant, and viral titers were calculated via the Spearman–Karber method.

### Comparison of the replication efficiency of mutant viruses in avian hosts

To compare the replication ability of major mammalian pathogenicity-related mutation (MPM) combinations with that of MVVs in avian hosts, 100 EID_50_ of each virus was mixed and inoculated into 10-day-old SPF ECEs. Allantoic fluid was harvested at 3 days post-inoculation and passaged two or three times. Viral RNA was extracted from allantoic fluid harvested from each passage along with the preinoculation virus mixture using the Patho Gene-spin DNA/RNA Extraction Kit (iNtRON Biotechnology), followed by complementary DNA (cDNA) synthesis (Enzynomics, Daejeon, Republic of Korea). Partial-length PB2 amplicons encompassing positions 66, 109, 133, 147, 339, 588, and 627 were subcloned into a TA cloning vector (Real Biotech Corporation, Taipei, Taiwan) and Sanger-sequenced to obtain per-clone haplotypes across all signature sites.

### Animal experiments

To assess the pathogenicity of MVVTTT_588_ in mice, 6-week-old female BALB/c mice were purchased from KOATEC (Pyeongtaek, Republic of Korea). Five BALB/c mice were intranasally inoculated with 10^6^ EID_50_/50 µL of each virus after anesthetization via an intraperitoneal injection of 15 mg/kg Zoletil 50 (Virbac, Carros, France). Negative control mice were intranasally injected with an equal volume of PBS. Mortality and weight loss were observed for 14 days, and mice that lost more than 20% of their initial weight were euthanized. To assess the replication efficiency of the virus in mouse lungs, six BALB/c mice were injected with PBS or 10^6^ EID_50_/50 μL of the mutant virus. The lungs were collected at 3 and 5 days post-inoculation (dpi) and stored at −80 °C until use. The lungs were ground using a TissueLyzer 2 (Qiagen, Hilden, Germany) with 5 mM stainless steel beads and a volume of PBS equal to 10% of the lung weight in suspension. Then, a tenfold volume of PBS was mixed with the ground tissues. After centrifugation at 3000 rpm for 10 min, the supernatants were used to measure viral titers.

### Viral quasi-species analysis

To assess the adaptive mutations present in the PB2 gene in viruses isolated from infected mouse lungs, viral RNA was extracted from 3 and 5 dpi lung homogenates. Then, the viral RNA was sequenced via the Illumina MiSeq method, as described previously, with modifications [30, 31]. Briefly, vRNA was amplified via reverse transcription (RT)-PCR with 0.2 μM MBTuni-12 and MBTuni-13 primers via the SuperScript IV one-step RT-PCR system (Invitrogen). All eight vRNA segments were amplified simultaneously and confirmed by gel electrophoresis of the PCR products. The sequences generated via Illumina MiSeq (BIONICS Co., Seoul, Republic of Korea) were analyzed using Geneious Prime (version 2023.2.1., Biomatters Ltd.). Paired reads were trimmed using the BBDuk script, followed by read merging. Sequences with lengths ranging from 150 to 290 were extracted and mapped to the reference sequence 01310 PB2. Variant analysis of mouse lungs was performed in compliance with methods published in a previous report [32].

### Sequence collection and analysis

For prevalence analysis of mutation combinations, PB2 protein sequences from avian (total 40,493 sequences), human (total 1762 sequences), and swine (total 12,420 sequences) influenza A viruses isolated between 1959 and 2023 were collected from the Global Initiative for Sharing All Influenza Data (GISAID) EpiFlu™ database [33]. Only complete sequences were included, and to focus on viruses of avian origin, PB2 gene sequences from H1 and H3 subtypes of human origin were excluded from the data collection and analysis. In contrast, swine H1 and H3 sequences were retained owing to frequent reassortment with avian influenza viruses and their relevance as mixing-vessel hosts at the avian–mammalian interface.

Sequences containing mutations other than the target mutations were excluded from the frequency analysis. For example, when analyzing MVV, sequences with MVV-147 T-588 T were excluded from the MVV frequency analysis. The sequences were aligned with the ClustalW multiple alignment tool of the BioEdit program (version 7.2.5), and the prevalence of major MPMs combinations was analyzed. For the prevalence analysis of the E627K mutation acquired during the mutational process of MVVTTT_588_, only PB2 protein sequences belonging to H5 viruses from avian and human hosts were included.

### Statistical analysis

Polymerase activity and viral titers were compared via one-way analysis of variance (ANOVA) analysis followed by Tukey’s multiple comparisons test and growth kinetics analysis in mammalian cell were compared via two-way ANOVA, followed by Tukey’s multiple comparisons test or Dunnett’s multiple comparisons test as described in the figure legends (GraphPad Prism version 10.0.2 for Windows, GraphPad Software, Boston, MA, USA). The results were considered statistically significant if *P* < 0.05.

### Ethics statement

All mouse experiments were carried out at the College of Pharmacy of Seoul National University (Seoul, Korea) following the recommendations of the National Institutes of Health’s Public Health Service Policy on the Humane Care and Use of Laboratory Animals (PHS Policy). The protocol was reviewed and approved by the Institutional Animal Care and Use Committee (IACUC) of Seoul National University (SNU-220407-3-2).

## Results

### Generation of recombinant PR8 strains harboring mutated 01310 PB2 genes and evaluation of competitive replication efficiency in ECEs

To evaluate the cumulative effects of different combinations of PB2 mutations on replication efficiency, we sequentially introduced mutations into the 01310 PB2 gene. Initially, the avian-prevalent MVV mutation (I66M/I109V/I133V; hereafter MVV) was generated, followed by stepwise addition of the I147T, K339T, and A588T (or E627K) mutations, resulting in the mutated PB2 variants MVVT_147_ (MVV/I147T), MVVTT_339_ (MVV/I147T/K339T), MVVTTT_588_ (MVV/I147T/K339T/A588T), and MVVTTK_627_ (MVV/I147T/K339T/E627K), respectively. These mutated genes were used to construct recombinant PR8 strains (rMVV, rMVVT_147_, rMVVTT_339_, rMVVTTT_588_, and rMVVTTK_627_) containing seven genome segments derived from PR8 and compared with the recombinant PR8 strain (rPR8) for replication efficiency in embryonated chicken eggs (ECEs) (Table 1).

**Table 1:** **Genomic constellations of recombinant PR8 viruses possessing different mutation combinations in PB2 genes and viral titers in embryonated chicken eggs (ECEs)**

Recombinant virus	Genomic constellation								Viral titer (logEID_50_/mL)
	PB2	PB1	PA	HA	NP	NA	M	NS	
rPR8	PR8	PR8	PR8	PR8	PR8	PR8	PR8	PR8	9.75 ± 0.20
rMVV	01310 (MVV)	PR8	PR8	PR8	PR8	PR8	PR8	PR8	9.92 ± 0.23
rMVVT_147_	01310 (MVVT_147_)	PR8	PR8	PR8	PR8	PR8	PR8	PR8	9.42 ± 0.31
rMVVTT_339_	01310 (MVVTT_339_)	PR8	PR8	PR8	PR8	PR8	PR8	PR8	7.92 ± 0.12 *^a^
rMVVTTT_588_	01310 (MVVTTT_588_)	PR8	PR8	PR8	PR8	PR8	PR8	PR8	9.92 ± 0.12
rMVVTTK_627_	01310 (MVVTTK_627_)	PR8	PR8	PR8	PR8	PR8	PR8	PR8	9.92 ± 0.12

^a^The statistical significance was analyzed by one-way ANOVA followed by Tukey’s multiple comparisons test. The asterisks indicate significant difference between the titers of rMVVTT339 and other viruses (*p < 0.0001)

Among these recombinants, rMVVTT_339_ presented significantly lower viral titers (up to approximately 100-fold) than the other recombinant strains did. No significant differences were detected among rMVV, rMVVTTT_588_, and rMVVTTK_627_, although rMVVT_147_ presented slightly lower viral titers than these strains did.

**Figure 1:**
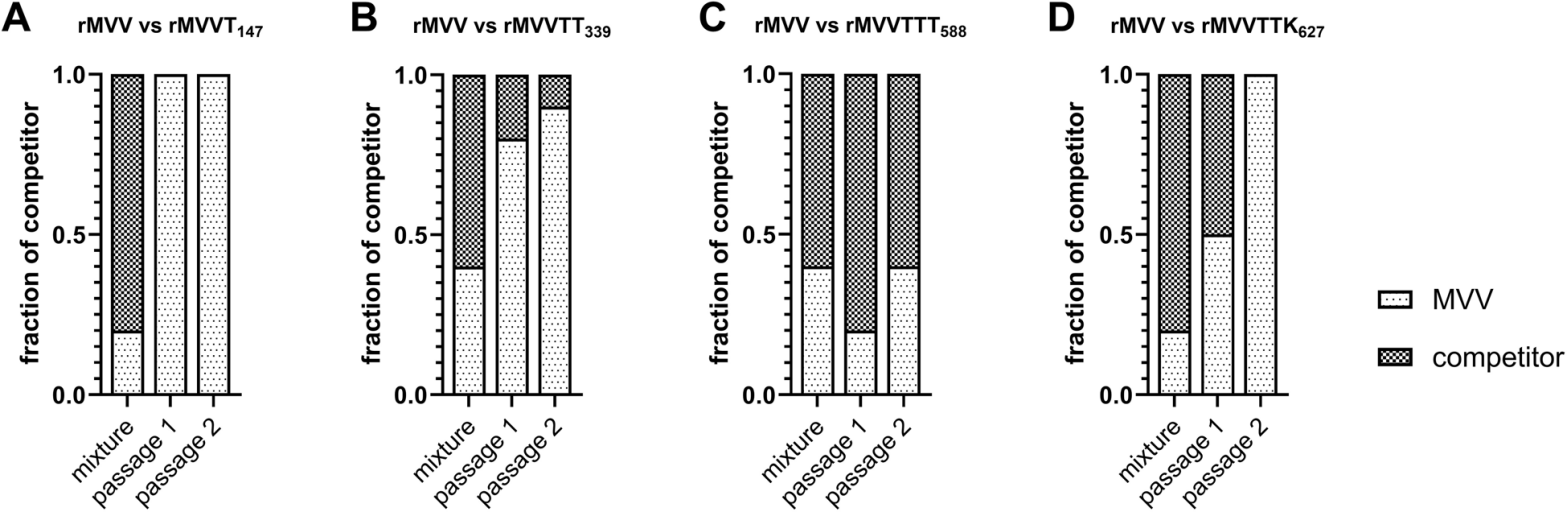
**Comparison of the replication efficiencies of recombinant PR8 strains via competitive replication assays in ECEs.** Equal amounts of recombinant PR8 viruses harboring MVV and MVVT_147_ (**A**), MVVTT_339_ (**B**), MVVTTT_588_ (**C**), and MVVTTK_627_ (**D**) were inoculated into 10-day-old SPF ECEs. Allantoic fluid harvested at 3 dpi were analyzed by cloning the amplified PB2 genes into the TA cloning vector. The population of PB2 genes was analyzed by sequencing each clone along with the pre-inoculation mixture.

Next, we assessed the competitive replication efficiency of the recombinant viruses by performing direct competition assays, in which rMVVT_147_, rMVVTT_339_, rMVVTTT_588_, and rMVVTTK_627_ individually competed against rMVV over two sequential passages in ECEs (Figure 1). Although the proportions of rMVVT_147_ and rMVVTTK_627_ were greater than those of rMVV in the initial virus mixtures, these strains rapidly disappeared following the first and second passage, respectively (Figures 1A, D). Similarly, the percentage of rMVVTT_339_ markedly decreased from 60% initially to 10% after two passages (Figure 1B). In contrast, the proportion of rMVVTTT_588_ was consistently maintained throughout the passages (Figure 1C), indicating superior competitive replication efficiency and compatibility with the PR8 gene segments. Collectively, these data demonstrate a clear hierarchy in competitive fitness among the tested PB2 mutants.

### Effects of PB2 mutations on polymerase activity in mammalian cells

**Figure 2:**
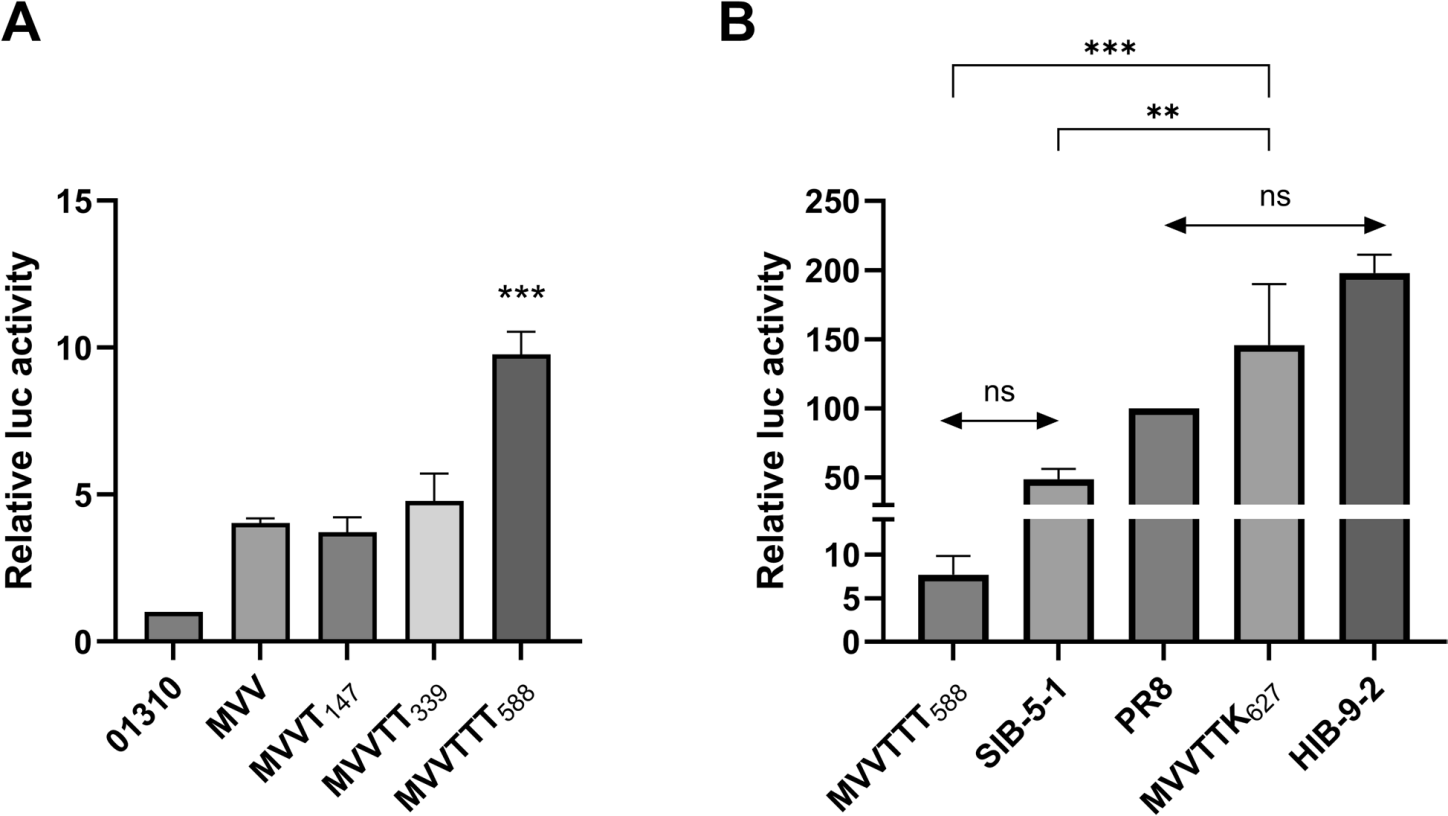
**Comparison of the effects of the combinations of mutations on polymerase activity in mammalian cell lines.** **A** The effects of the accumulated mutations on polymerase activity were evaluated in comparison with that of PB2 genes that possess only the minimal essential mutations (MVVs). The data were normalized as the percentage of polymerase activity relative to that of the wild-type 01310 PB2 gene. Asterisks indicate the differences between MVVTTT_588_ and other mutations. **B** The effects of MVVTTT_588_ and MVVTTK_627_ on polymerase activity were evaluated in comparison with 01310 PB2 genes containing previously characterized PB2 mutations from pandemic 2009 (SIB-5-1) and human seasonal influenza viruses (HIB-9-2). The data were normalized as the percentage of polymerase activity relative to that of the PR8 PB2 gene. Statistical significance was analyzed by one-way ANOVA followed by Tukey’s multiple comparisons test (**p < 0.01, ***p < 0.001). Each experiment was performed independently, and the displayed data are the average of triplicate data from one experiment.

We assessed the effects of PB2 mutations on polymerase activity via mini-genome assays in mammalian 293T cells (Figure 2). First, we compared the polymerase activities of the parent 01310 PB2 gene with those of the mutated variants MVV, MVVT_147_, MVVTT_339_, and MVVTTT_588_ (Figure 2A). As expected, 01310 presented the lowest polymerase activity, whereas MVV, MVVT_147_, and MVVTT_339_ presented similarly modest increases in polymerase activity without statistically significant differences, suggesting minimal contributions from I147T and I147T-K339T. However, compared with the other variants, MVVTTT_588_ displayed significantly increased polymerase activity.

Next, as benchmarks for mammalian-adapted polymerase activities, we included the 01310 PB2 gene containing previously characterized PB2 mutations from pandemic 2009 (MVV/147T/271A/588T/590S/591R; hereafter SIB-5-1) and human seasonal influenza viruses (MVV/9N/199S/271A/526R/588T/590S/627K/674T/702R; hereafter HIB-9-2), as well as PR8 PB2 (Figure 2B) [13, 34]. While MVVTTT_588_ showed polymerase activity comparable to that of SIB-5-1, MVVTTK_627_ exhibited significantly greater activity, similar to PR8 PB2 and HIB-9-2. Although the multiple co-occurring PB2 substitutions in PR8 PB2 and HIB-9-2, as well as the MVV-I147T-K339T background of MVVTTK_627_, should be considered, these comparisons suggest that inclusion of E627K within these mutation constellations is associated with increased activity in mammalian cells.

### Effects of PB2 mutations on the replication efficiency of recombinant PR8 strains in mammalian cells

**Figure 3:**
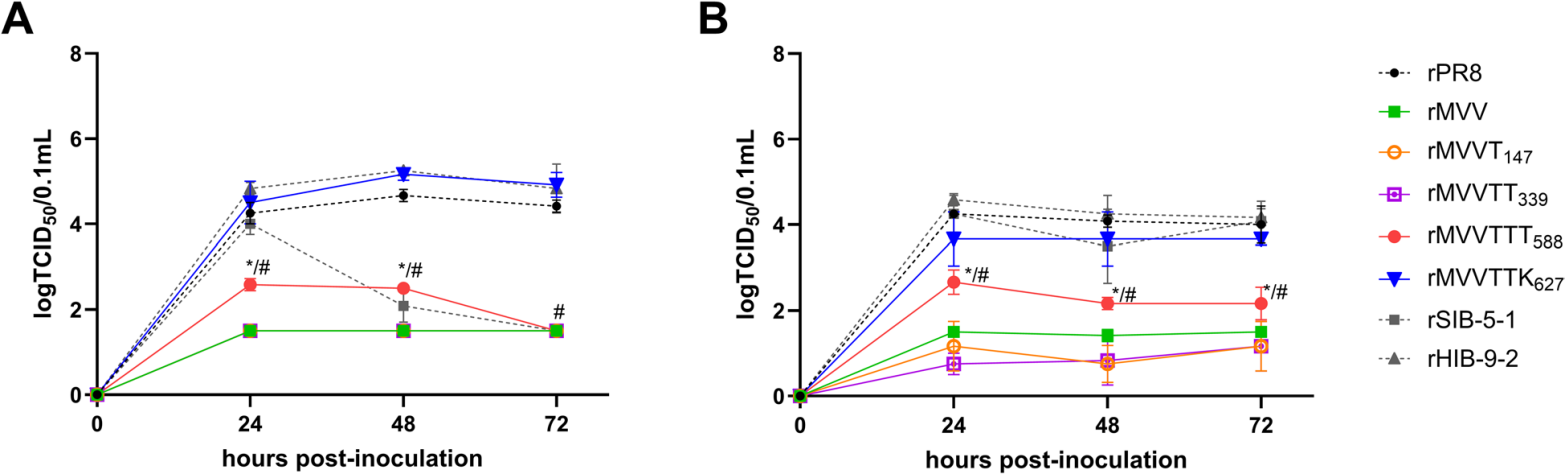
**Effect of the combinations of mutations on replication efficiency of recombinant PR8 viruses in mammalian cell lines.** The MDCK (**A**) and Calu-3 (**B**) cell lines were inoculated with recombinant viruses at an MOI of 0.001, and the supernatants were collected at 0, 24, 48, and 72 h post-inoculation. The statistical significance of differences between the recombinant viruses was analyzed via ordinary two-way ANOVA followed by Tukey’s multiple comparisons test. The differences between MVVTTT_588_ and MVV, MVVT_147_, and MVVTT_339_ (*p < 0.05) and between MVVTTT_588_ and MVVTTK_627_, rPR8, and rHIB-9-2 (#*p* < 0.05) at each time point were determined (**A**, **B**). However, no significant differences were detected between MVVTTT_588_ and MVV at 48 and 72 h post-infection in Calu-3 cells.

We further analyzed the replication efficiencies of recombinant PR8 strains containing mutated PB2 genes in the mammalian MDCK and Calu-3 cell lines, comparing rMVV, rMVVT_147_, rMVVTT_339_, rMVVTTT_588_, rMVVTTK_627_, and rPR8 as well as with rSIB-5-1 and rHIB-9-2, which are PR8-derived strains containing the SIB-5-1 and HIB-9-2 PB2 genes, respectively (Figure 3). Consistent with the results of the mini-genome assay, rMVVTTK_627_ presented the highest viral titers among all recombinant viruses harboring PB2 variants, comparable to those of rSIB-5-1, rHIB-9-2, and rPR8 at all time points, significantly surpassing those of the other variants.

Moreover, rMVV, rMVVT_147_, and rMVVTT_339_ consistently presented the lowest viral titers across all time points, with no significant differences among these three strains. Notably, the titers of rMVVTTT_588_ were significantly greater than those of rMVV, rMVVT_147_, and rMVVTT_339_ at most time points in both cell lines. Overall, these results indicate that the I147T and I147T-K339T mutations confer minimal advantages for mammalian replication, whereas the combined triad I147T-K339T-A588T substantially enhances viral replication efficiency in mammalian cells, although less effectively than the variant containing E627K.

### The pathogenicity of rMVVTTT_588_ is reduced compared with that of the parental PR8 strain in mice

**Figure 4:**
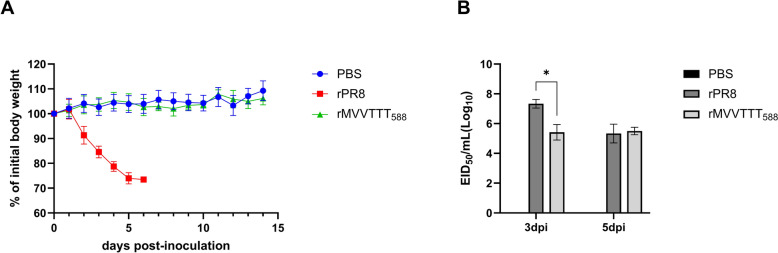
**The effect of MVVTTT**_**588**_** on pathogenicity in mice. The effect of MVVTTT**_**588**_** on pathogenicity in mice was evaluated by monitoring body weight changes** (**A**) and measuring viral titers in the lungs (**B**). Eleven 6-week-old BALB/c mice per group were challenged with 1 × 10^6^ EID_50_ of each virus or with PBS. For five mice, weight changes were observed for 14 days, and mice that experienced more than 25% weight loss were euthanized. Three mice each were euthanized at 3 and 5 days post-infection, and their lungs were harvested for evaluation of viral loads. Statistical significance was analyzed by one-way ANOVA followed by Dunnett’s multiple comparisons test (***p < 0.001, ****p < 0.0001).

We next assessed the potential mammalian pathogenic risk of MVVTTT_588_ in vivo, as rMVVTTT_588_ demonstrated moderate replication efficiency in mammalian cells with strong competitiveness in ECEs. Six-week-old BALB/c mice were inoculated intranasally with 10^6^ EID_50_ of either rMVVTTT_588_ or rPR8, which served as a virulent control (Figure 4). Mice inoculated with rPR8 exhibited severe body weight loss within 4 days post-inoculation, whereas those inoculated with rMVVTTT_588_ maintained stable body weights comparable to those of the PBS-inoculated control over the 14-day period (Figure 4A).

The lung viral titers of the rPR8-inoculated mice at 3 and 5 days post-infection (dpi) reached 10^6.6^EID_50_/0.1 mL and 10^4.6^EID_50_/0.1 mL, but those of the rMVVTTT_588_-inoculated mice were 10^4.6^EID_50_/0.1 mL and 10^4.6^EID_50_/0.1 mL, respectively (Figure 4B). Thus, rMVVTTT_588_ replicated less efficiently than did rPR8 at 3 dpi but presented similar viral titers at 5 dpi.

To investigate the potential mechanisms underlying this change at 5 dpi, deep sequencing analysis of the PB2 gene was performed on lung homogenates collected at 3 and 5 dpi. Six novel mutations, viz. E627K, D701N, T521K, D195N, R753K/G, and L648V, were identified (Table 2). Notably, E627K emerged consistently in all the samples (6/6), with frequencies varying widely from 0.11% to 73.11%. Additionally, D701N was observed in five samples (5/6), with frequencies ranging from 0.5% to 8.6%, and T521K was observed in three samples (3/6), with frequencies between 4.8% and 13.9%. D195N and R753K/G were detected in two samples (2/6), with frequencies of 5.4% and 17.0%, respectively, for D195N and 3.0% and 4.5%, respectively, for R753K/G. L648V was detected in only one sample, with a frequency of 4.5%. Interestingly, the frequency of T521K surpassed that of E627K in mouse E at 5 dpi (13.9% versus 0.1%), whereas the E627K mutation was the dominant mutation in the remaining five samples. All the mice presented at least two mutations, and mouse A (3 dpi) presented all six mutations simultaneously, highlighting rapid and heterogeneous within-host viral evolution. These findings indicate that, while the I147T–K339T–A588T triad alone is attenuated in mice, it provides a genetic background that leads to additional acquisition of key mammalian-adaptation markers such as E627K, closing the replication gap within 2 days and thereby increasing zoonotic risk.

**Table 2:** **Frequencies of mammalian adaptation-related mutations (MAMs) of rMVVTTT**_**588**_ **in mouse lungs**

MAM	3 dpi			5 dpi			Mice with mutation (*n*/6)^b^
	Mouse A	Mouse B	Mouse C	Mouse D	Mouse E	Mouse F	
E627K	**73.1 **(348/476)^a^	**55.7** (44/79)	**25.0** (8/32)	**49.2** (409/832)	0.1 (1/931)	**9.5** (33/348)	6/6
D701N	2.4 (11/466)	3.9 (10/256)	4.6 (25/549)	0.5 (6/1068)	nd ^c^	8.6 (129/1495)	5/6
T521K	8.2 (7/85)	nd	nd	4.8 (8/168)	**13.9** (36/260)	nd	3/6
R753K/G	3.0 (6/200) ^d^	4.5 (7/156)^e^	nd	nd	0.15 (1/663)^d^	nd	3/6
D195N	17.0 (8/47)	nd	nd	nd	nd	5.4 (8/148)	2/6
L648V	8.2 (32/390)	nd	nd	nd	nd	nd	1/6

^a^Numbers in parentheses show variant reads/total reads, and highest frequency per mouse is bolded

^b^Number of mice (out of six) in which the mutation is present at ≥ 0.1%

^c^Not detected

^d^R753K; ^e^R753G

Please provide a definition for the significance of [bold] in the table.As stated in the table footnotes, the highest frequency per mouse is bolded ( ^a^ in table footnotes).

### Recurrent acquisition of E627K at every evolutionary point of MVVTTT_588_

**Figure 5:**
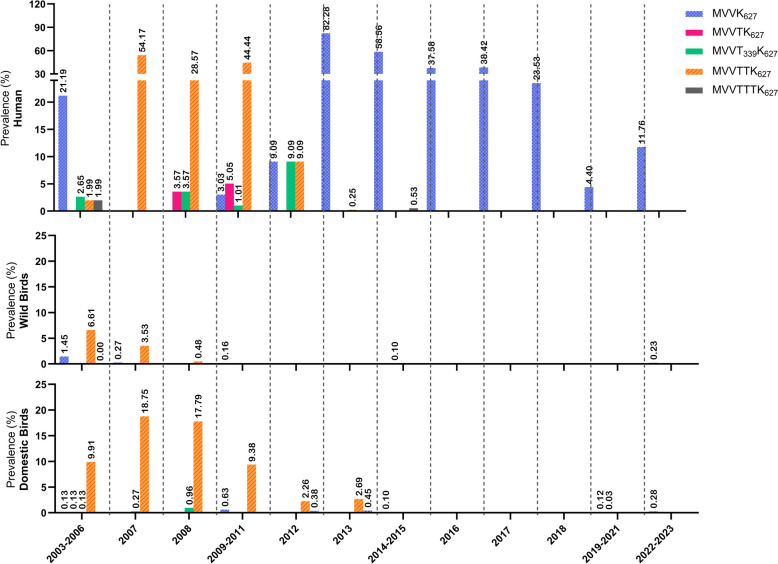
**Acquisition of the E627K mutation among human and avian hosts during the stepwise accumulation of the I147T, K339T, and A588T mutations in H5 viruses.** Years are fractionated according to the Chinese vaccination program, as depicted in Additional file 1 (2003–2006: N-28, Re-1; 2007: Re-1, Re-4; 2008: Re-1, Re-4, Re-5; 2009–2011: Re-4, Re-5; 2012: Re-4, Re-5, Re-6; 2013: Re-4, Re-6; 2014–2015: Re-6, Re-7; 2016: Re-6, Re-7, Re-8; 2017: Re-6, Re-8; 2018: Re-8; 2019–2021: Re-11, Re-12; 2022–2023: Re-13, Re-14). All the analyzed sequences were collected from the GISAID database on 2024.10.14, and only complete PB2 sequences of H5 viruses were retrieved.

The early emergence and high frequency of E627K at 3 dpi suggest that this substitution increases the lung viral load in mice. To understand how E627K becomes incorporated into circulating H5Nx viruses, we next analyzed its prevalence among human and avian hosts across sequential PB2 backgrounds that accumulate I147T, K339T, and A588T (Figure 5). Given that large-scale vaccination significantly impacts influenza virus evolution, we segmented the temporal analysis according to changes in the recommended H5Nx vaccination program, which has been ongoing since 2003 [35–37].

Across all evolutionary points, E627K consistently appeared more frequently in human isolates than in avian isolates. Notably, MVVTTK_627_ exhibited a relatively high frequency in avian hosts between 2003 and 2006 but declined thereafter. From 2007 onward, its prevalence was greater in human isolates than in avian isolates. Similarly, MVVK_627_ remained sporadic in birds but persisted in humans from 2009 onward. Whereas MVVTTK_627_ and MVVK_627_ rose sequentially and became prevalent, MVVTTTK_627_ remained rare and was detected only sporadically in domestic birds.

Within avian hosts, E627K-containing variants were detected more often in domestic birds than in wild birds. Specifically, MVVTTK_627_ occurred more commonly in domestic birds from 2003 to 2013 (2.26–18.75%), whereas MVVK_627_ was prevalent from 2009 to 2011 (0.63%) and 2019–2023 (0.12–0.28%) (Figure 5, see Additional file 1). Notably, beginning in 2016, the frequencies of MVVTK_627_, MVVT_339_K_627_, MVTTK_627_, and MVVTTTK_627_ declined significantly in both human and avian hosts.

### First appearance, frequency, and distribution of single and combined mammalian-adaptive mutations in influenza A viruses from different hosts

**Figure 6:**
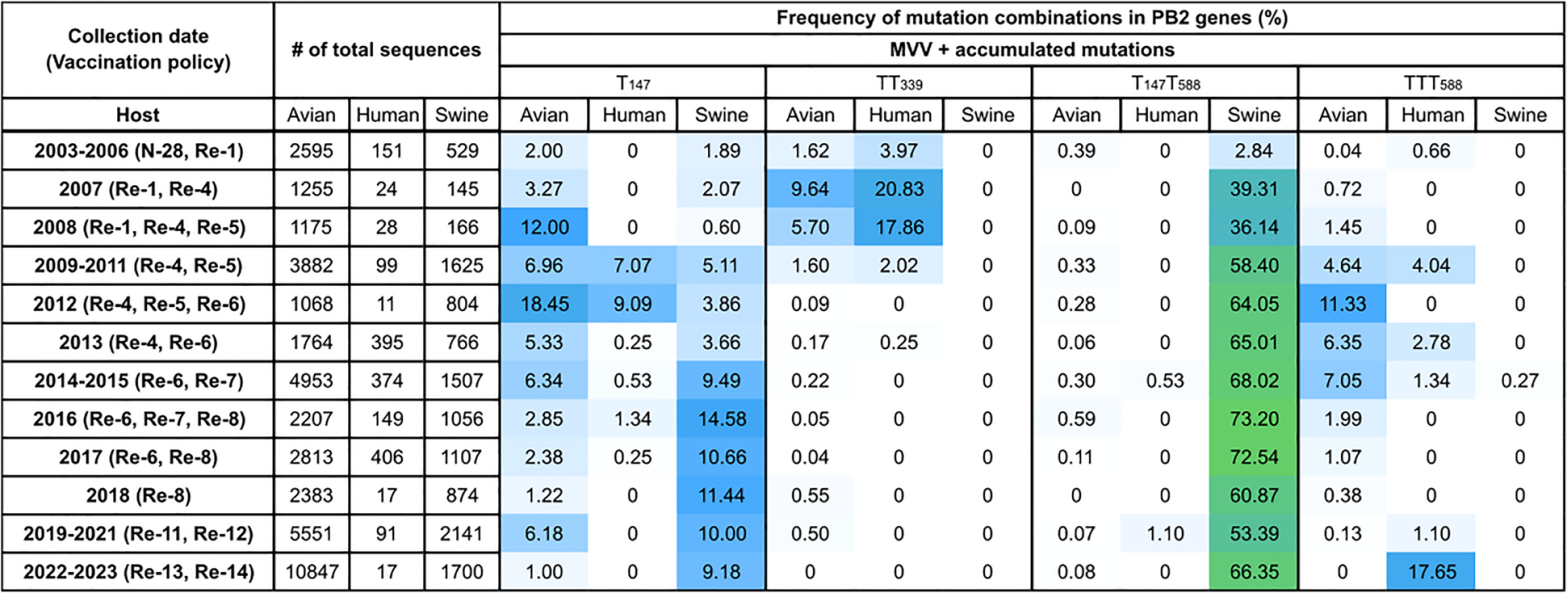
**Heatmap representation of the prevalence of the mutations in avian, swine, and human hosts.** The frequencies of single and combined PB2 mutations in avian, swine, and human hosts were compared and visualized via a heatmap, as follows: 0 (white), 30% (blue), 100% (green). All the analyzed sequences were collected from the GISAID database on 14 October 2024, and human H1 and H3 subtypes were excluded from the analysis.

To clarify the emergence and host distribution patterns of mutations comprising MVVTTT_588_, we analyzed the frequency and distribution of single and combined PB2 mutations (I147T, K339T, and A588T) in influenza A viruses (IAVs) isolated from different host species (Figure 6, see Additional file 2).

All analyzed mutations were initially detected prior to or during 2003 (see Additional file 2). The I147T mutation was first identified in wild bird-derived AIVs from Canada in the late 1970s and was later identified in poultry populations from the USA and England. Although predominantly detected among wild birds, this mutation was also identified in swine hosts in 1989, after prior detection in North American migratory bird H1N1 isolates, clearly indicating its avian origin. Interestingly, the frequency of MVVT_147_ has increased among swine hosts since 2009, surpassing that of avians from 2014 onward, suggesting that MVVT_147_ has no negative fitness impact on swine hosts (Figure 6).

The K339T mutation was first observed in avian H5N1, human H5N1, and swine H3N2 viruses in 2003 (see Additional file 2). Although initially abundant in avian isolates, this mutation displayed consistently higher frequencies in human isolates, predominantly of the H5N1 subtype, from 2003 to 2014. A similar trend was observed for the combined mutation MVVTT_339_, which emerged initially in avian hosts and subsequently became prevalent in humans from 2003 to 2008, demonstrating its importance in mammalian adaptation.

The earliest detection of A4588T (MVVT_588_) was traced back to poultry in Scotland in 1959, with sporadic isolates subsequently detected in North American wild birds during the 1970s and 1980s (see Additional file 2). In 1992, the mutation emerged in swine H3N2 viruses, followed by avian H2N2 isolates in North America. Unlike MVVTT_339_, the double mutation MVVT_147_T_588_ was predominantly identified among North American swine viruses and became highly prevalent in swine hosts from 2007 onward, maintaining a persistent presence.

For the MVVTTT_588_ triad mutation, the prevalence increased notably in avian hosts from 2009 to 2015 following the peak of MVVTT_339_ in 2007, as did that in humans (Figure 6). After a transient decline from 2016 to 2018, MVVTTT_588_ reemerged as a human virus in 2020 and reached its highest prevalence from 2022 to 2023 (17.65%), all within clade 2.3.2.1c H5N1 HPAIVs. The concurrent fluctuations in avian and human viruses and recent increase in prevalence underscore the potential for increased risk of poultry-to-human transmission.

### Generation of recombinant clade 2.3.2.1c H5N1 strains harboring mutated PB2 genes and evaluation of their replication efficiency in ECEs

The observed prevalence and sequential accumulation of I147T and K339T mutations among avian isolates contrasted with the lower replication efficiencies of rMVVT_147_ and rMVVTT_339_ than of rMVV in ECEs (Table 1, Figure 1) [34]. Since optimal viral replication depends heavily on the compatibility of PB2 mutations with HA and NA genes, we assessed whether these mutations affected replication when combined with HA and NA from clade 2.3.2.1c H5N1 viruses [38]. Recombinant viruses harboring HA and NA genes from the clade 2.3.2.1c H5N1 strain (K10), internal genes from PR8, and mutated PB2 genes (rK10-MVV, rK10-MVVT_147_, rK10-MVVTT_339_, and rK10-MVVTTT_588_) were generated and evaluated for growth efficiency in ECEs (Table 3). Owing to biosafety concerns, the multiple basic amino acids at the HA cleavage site were removed, and rK10-MVVTTK_627_ was not generated.

**Table 3:** **Genomic constellations of recombinant clade 2.3.2.1c H5N1 strains possessing different PB2 mutation combinations and viral titers in embryonated chicken eggs (ECEs)**

Recombinant virus	Genomic constellation								Viral titer (logEID_50_/mL)
	PB2	PB1	PA	HA	NP	NA	M	NS	
rK10-P	PR8	PR8	PR8	K10-483	PR8	K10-483	PR8	PR8	8.65 ± 0.11^**a^
rK10-MVV	01310 (MVV)	PR8	PR8	K10-483	PR8	K10-483	PR8	PR8	9.75 ± 0.35
rK10-MVVT_147_	01310 (MVVT_147_)	PR8	PR8	K10-483	PR8	K10-483	PR8	PR8	9.33 ± 0.24
rK10-MVVTT_339_	01310 (MVVTT_339_)	PR8	PR8	K10-483	PR8	K10-483	PR8	PR8	9.25 ± 0.20
rK10-MVVTTT_588_	01310 (MVVTTT_588_)	PR8	PR8	K10-483	PR8	K10-483	PR8	PR8	9.07 ± 0.26

^a^Statistical significance was analyzed by one-way ANOVA followed by Dunnett’s multiple comparisons test. Asterisks indicate significant differences compared with the titer of rK10(MVV) (**p < 0.01)

The recombinant virus rK10-P, containing all internal genes of PR8, presented a significantly lower titer than did rK10-MVV, which was consistent with previous observations of the PR8-based clade 2.3.2.1c H5N1 vaccine strain [39]. In contrast, the viruses rK10-MVV, rK10-MVVT_147_, and rK10-MVVTTT_588_ yielded viral titers above 10^9.0^EID_50_/mL. Notably, the titer of rK10-MVVTT_339_ was approximately tenfold greater than that of the corresponding PR8-based rMVVTT_339_ and was not significantly different from that of high-yielding recombinant K10 strains (Table 3).

**Figure 7:**
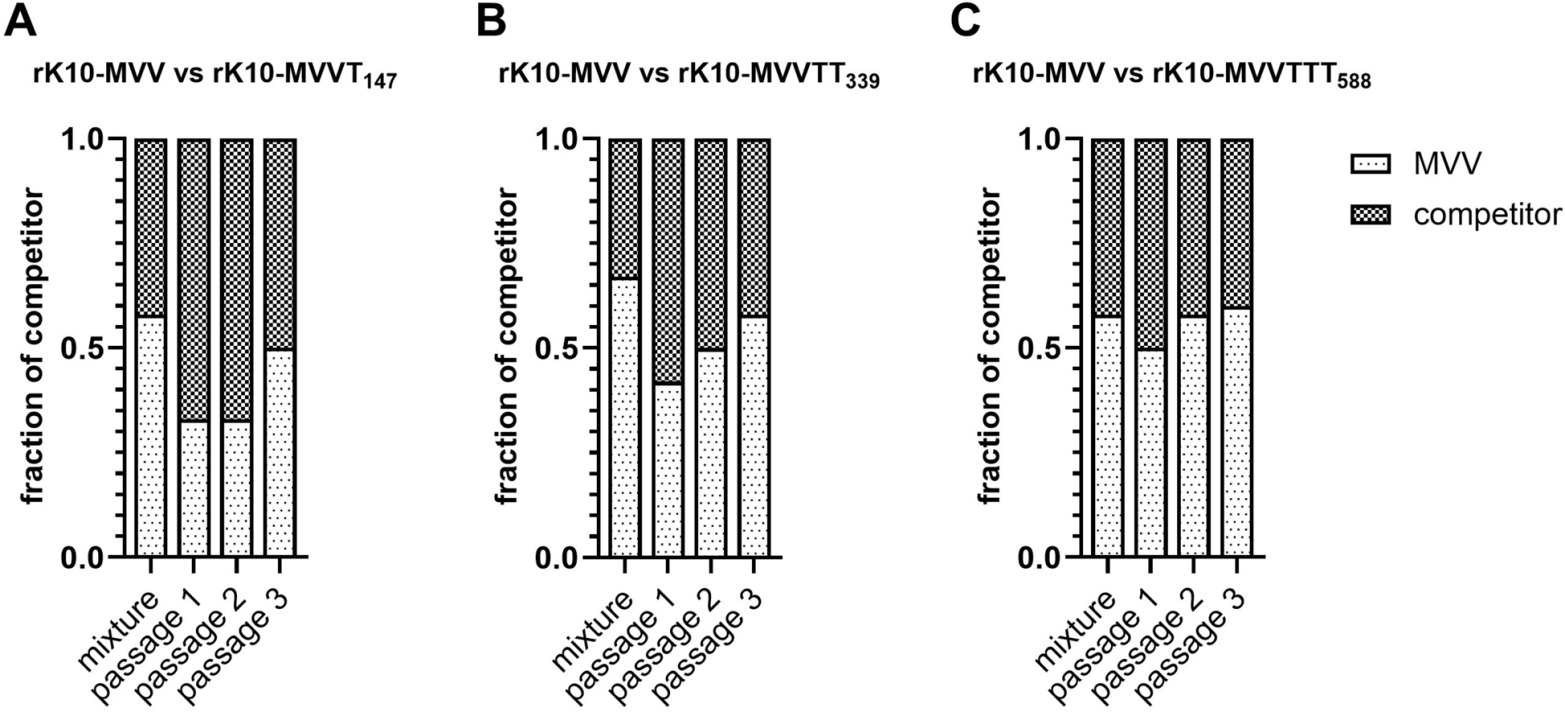
**Comparison of the replication efficiencies of recombinant clade 2.3.2.1c H5N1 strains via competitive replication assays in ECEs.** Proportions of PB2 quasispecies during passaging in ECEs. Equal amounts of recombinant K10-483 viruses harboring different MPM combinations were inoculated into 10-day-old SPF ECEs. Allantoic fluid harvested at 3 dpi were analyzed by cloning the amplified PB2 genes into the TA cloning vector. The population of PB2 genes was analyzed by sequencing each clone along with the pre-inoculation mixture.

This change in replication efficiency was also observed in competitive replication assays (Figure 7). In contrast to the corresponding recombinant PR8 strains, rMVVT_147_, rMVVTT_339_, rK10-MVVT_147_, and rK10-MVVTT_339_ coexisted with rK10-MVV until the end of three passages in ECEs, even maintaining proportions higher than those in the starting mixtures (Figures 7A, B). rK10-MVVTTT_588_ retained replication characteristics similar to those of its PR8-based counterpart (Figure 7C), highlighting its consistently advantageous replication profile across viral backbones.

### Replication efficiencies of clade 2.3.2.1c H5N1 strains harboring mutated PB2 genes in mammalian cells

**Figure 8:**
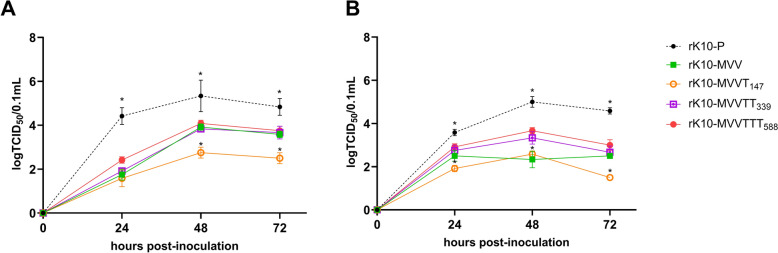
**Effects of the investigated mutations on the replication efficiency of recombinant clade 2.3.2.1c H5N1 strains in mammalian cell lines.** Effects of MPM combinations on the growth kinetics of recombinant clade 2.3.2.1c H5N1 viruses in mammalian cell lines. MDCK (**A**) and Calu-3 (**B**) cell lines were inoculated with recombinant viruses at an MOI of 0.001, and the supernatants were collected at 0, 24, 48, and 72 h post-inoculation. Statistical significance was analyzed via ordinary two-way ANOVA followed by Tukey’s multiple comparisons test. The differences between rK10-P, rK10-MVVT_147_, and other viruses are indicated with asterisks (*p < 0.001).

The replication efficiencies of rK10, rK10-MVV, rK10-MVVT_147_, rK10-MVVTT_339_, and rK10-MVVTTT_588_ were compared in MDCK and Calu-3 cells (Figure 8). Compared with those of other viruses, the titers of the rK10-P virus were significantly greater at most time points in both MDCK and Calu-3 cells. rK10-MVV, rK10-MVVTT_339_, and rK10-MVVTTT_588_ presented similar viral titers in MDCK cells at 48 and 72 h post-inoculation (hpi), whereas rK10-MVVT_147_ presented significantly lower titers (Figure 8A). In Calu-3 cells, rK10-MVVTT_339_ and rK10-MVVTTT_588_ displayed comparable replication efficiencies, with both showing significantly higher viral titers than rK10-MVV at all time points (Figure 8B). Collectively, these results demonstrate the enhanced compatibility of MVVTT_339_ in the genetic background of the clade 2.3.2.1c H5N1 virus and that the MVVTTT_588_ mutation increases replication efficiency in both avian and mammalian host systems regardless of subtype.

### Coevolution of the HA and PB2 genes in clade 2.3.2 and clade 2.3.2.1 H5N1 viruses

We observed clear differences in the viral characteristics between recombinant PR8 and K10 viruses harboring MVVT_147_, MVVTT_339_, and MVVTTT_588_. To understand the coevolutionary relationships among the HA, NA, and PB2 genes, we analyzed sequence data from clade 2.3.2 viruses and their subclades (2.3.2.1, 2.3.2.1a, 2.3.2.1b, and 2.3.2.1c) isolated between 2005 and 2012, focusing on key mutations predetermined to impact the activities of HA, NA, and PB2 (Table 4). Specifically, the frequencies of HA mutations (a 144N-glycosylation-inducing mutation, 144NG, and V223I), NA mutations (a 20-amino-acid deletion), and PB2 mutations (MVVT_147_, MVVTT_339_, and MVVTTT_588_) were compared. Nearly all the isolates carried the NA-stalk deletion except for a few wild bird-derived clade 2.3.2 viruses, so subsequent analyses focused on the relationships between HA and PB2 mutations.

**Table 4:** **Mutation accumulation patterns of the HA and PB2 genes of clade 2.3.2 and 2.3.2.1 H5Nx AIVs**

Gene (no. of seq)	Frequency of mutation (%)																
HA	None				V223I				144NG^a^				144NG + V223I				Total
PB2	MVV	T_147_^b^	TT_339_^b^	TTT_588_^b^	MVV	T_147_	TT_339_	TTT_588_	MVV	T_147_	TT_339_	TTT_588_	MVV	T_147_	TT_339_	TTT_588_	
2.3.2 (68)	60.3	0	5.88	0	0	0	0	0	10.29	0	0	0	0	0	0	0	76.47
2.3.2.1 (34)	0	2.94/2.94^c^	0	5.88	0	0	0	0	0	2.94/2.94^c^	0	82.35	0	0	0	0	94.11
2.3.2.1a (246)	0	0	0	0	0	0	1.63	4.07	0	0	0.41	0	0	4.07/2.94^c^	39.84	47.97	97.99
2.3.2.1b (48)	0	0	0	0	0	0	0	0	6.25	0	0	93.75	0	0	0	0	100
2.3.2.1c (142)	1.41	0	0	0	0	0	0	3.52	0	0	0	0.70	0	0.70	2.11	87.32	95.76

^a^144NG, mutation resulting in 144 N-glycosylation

^b^Additional mutations of MVV

^c^Frequencies of MVVT_147_/MVVT_147_T_588_

Most clade 2.3.2 viruses isolated in 2005 lacked HA or PB2 mutations (60.3%), with only minor occurrences of MVVTT_339_ (5.88%) or 144NG mutations (10.29%). However, clade 2.3.2.1 viruses, which emerged in 2007, predominantly acquired the 144NG mutation along with PB2 mutations. A total of 82.35% carried MVVTTT_588_, whereas smaller fractions possessed MVVT_147_ or MVVT_147_T_588_ mutations (each 2.94%). Only a small proportion of clade 2.3.2.1 viruses carried MVVTTT_588_ (5.88%) or MVVT_147_/MVVT_147_T_588_ (2.94%/2.94) mutations without accompanying HA mutations. This finding indicates that, as clade 2.3.2 diverged into clade 2.3.2.1 and acquired 144NGS, PB2 also underwent stepwise accumulation of mutations.

All clade 2.3.2.1b viruses acquired the 144NG mutation, with 93.75% also harboring the MVVTTT_588_ mutation. Most clade 2.3.2.1a and 2.3.2.1c viruses acquired both 144NG and V223I mutations (91.88% and 90.13%, respectively), although they presented different patterns of additional PB2 mutations. Clade 2.3.2.1a viruses with 144NG and V223I mutations presented a slightly greater frequency of MVVTTT_588_ (47.97%) than MVVTT_339_ (39.84%). In contrast, clade 2.3.2.1c viruses presented a much greater prevalence of MVVTTT_588_ (87.32%) than MVVTT_339_ (2.11%), suggesting an optimal balance between the HA of clade 2.3.2.1c viruses and PB2 carrying MVVTTT_588_.

### Effect of vaccine programs on the spread of the MVVTTT_588_ mutation across different clades of H5Nx viruses and different countries

**Figure 9:**
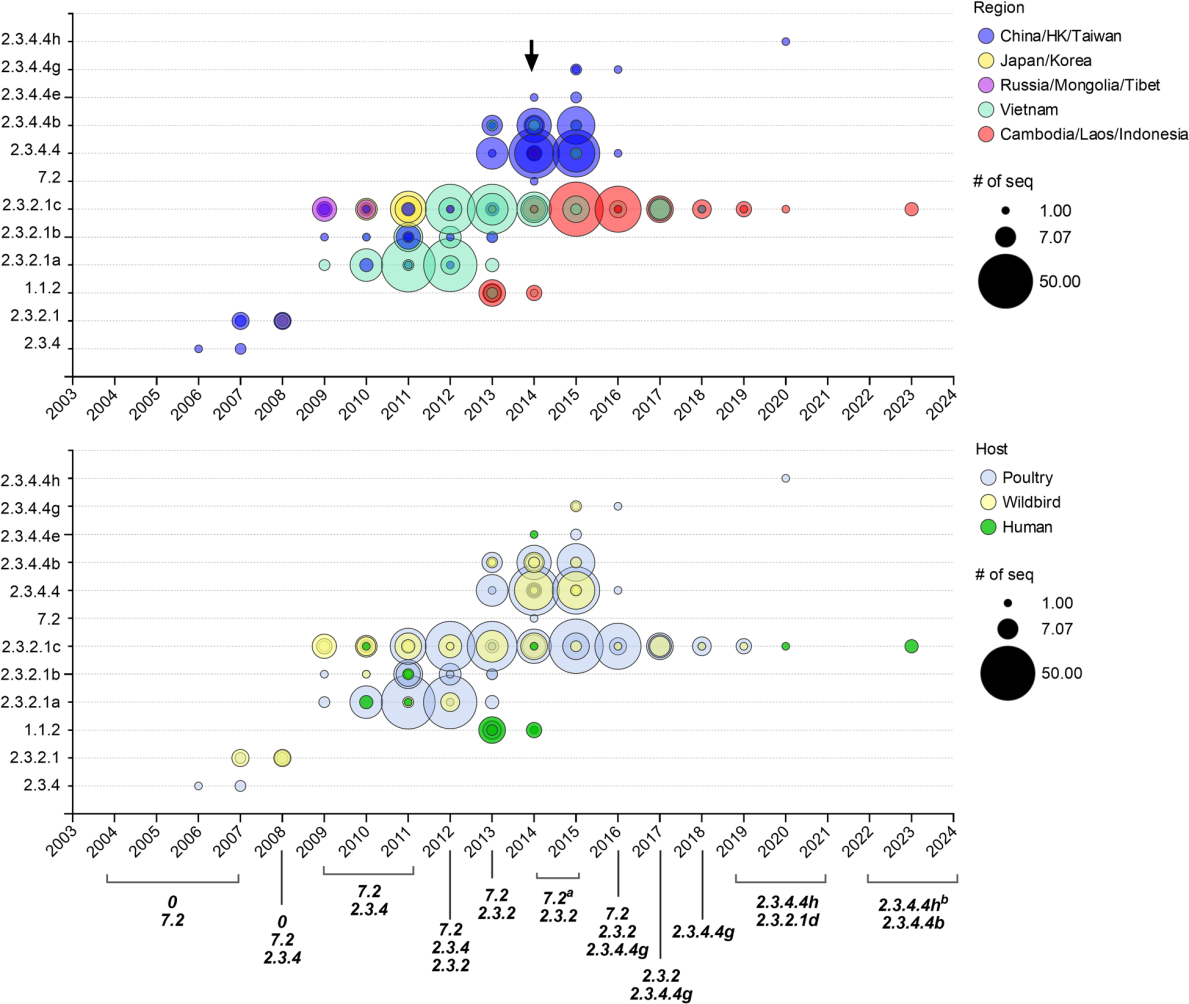
**Geographical and clade distributions of viruses harboring MVVTTT**_**588**_. H5 viruses harboring the MVVTTT_588_ combination were analyzed according to their clade, geographic origin (above), and host (below). The labels beneath the below graph list the clade designations for vaccine strains reported to be implemented in each year. The clades of the viruses are classified as they were assigned in the GISAID database, except clade 2.3.4.4b. To distinguish them from recently circulating clade 2.3.4.4b viruses, clade 2.3.4.4b H5N6 viruses isolated from 2012 to 2013 were classified as clade 2.3.4.4. The arrow indicates the initiation of the Re-6 vaccine targeting 2.3.2.1 recommended in Vietnam. ^a^Vaccines targeting clade 7.2 viruses changed from Re-4 to Re-7. ^b^Vaccines targeting clade 2.3.4.4 h viruses changed from Re-11 to Re-13.

To elucidate the dissemination patterns of MVVTTT_588_ across various H5Nx clades and regions with distinct vaccination strategies, avian and human H5Nx isolates harboring mutations collected between 2003 and 2023 were analyzed (Figure 9). MVVTTT_588_ first appeared in poultry-derived clade 2.3.4 H5N1 viruses in 2006–2007. The mutation subsequently emerged in clade 2.3.2.1 H5N1 viruses from poultry and wild birds in China (2007–2008) with high PB2 genetic identity (99.2–99.7% amino acid identity) to earlier clade 2.3.4 viruses, suggesting interclade PB2 gene transfer in China (see Additional file 3).

Since 2009, MVVTTT_588_ has spread among clades 2.3.2.1a, b, and c viruses across poultry populations in Vietnam, China, Russia, and Mongolia. Clades 2.3.2.1a and 2.3.2.1b harboring the mutation largely vanished after 2014, whereas clade 2.3.2.1c viruses persisted primarily in Vietnam, apart from a transient resurgence in 2017. Since 2015, MVVTTT_588_-carrying clade 2.3.2.1c viruses have spread to neighboring Cambodia and Laos. Moreover, the detection of MVVTTT_588_ in China resurged within clade 2.3.4.4 and its subclades in 2013.

### Probable emergence of diverse H5Nx HPAIVs under immune pressure exerted by H5N1 vaccination

While our analysis of key mutations in HA and PB2 in clade 2.3.2.1 and its subclades revealed a coevolutionary relationship between these segments (Table 4), the first identification of triad mutations in clade 2.3.4 in 2006, prior to their appearance in clade 2.3.2.1, prompted further analysis of the PB2 gene within clade 2.3.4 viruses. MVVTT_339_ was prevalent among clade 2.3.4 H5N1 viruses isolated in 2005 (53.33%) prior to the first identification of MVVTTT_588_ in 2006 (3.33%), which was consistent with the stepwise acquisition of A588T in poultry (see Additional file 4).

To trace the parallel evolution of surface genes, we analyzed the HA and NA segments of clade 2.3.4 H5N1, H5N5, and H5N2 viruses and clade 2.3.4.4 H5N2 and H5N8 viruses from 2005 to 2012. Most of the clade 2.3.4 H5N1 viruses isolated from 2005 to 2006 presented a short N1 (a 20 amino acid deletion) and NGS at residue 158 (a 158N-glycosylation-inducing mutation, 158NG) in HA. A single outlier, A/goose/Yunnan/3798/2006 (YN/98), combined short-stalk N1 and PB2 with MVVTT_339_ but lacked 158NG (see Additional file 5).

## Discussion

In this study, we assessed the mammalian pathogenic potential conferred by the stepwise accumulation of four PB2 mutations (I147T, K339T, and A588T or E627K). Notably, I147T–K339T–A588T increased polymerase activity and replication in mammalian cells without compromising avian fitness. Sequence analysis revealed that these mutations emerged first in avian hosts and expanded under sustained H5 vaccination, suggesting that vaccine-mediated immune pressure is a key extrinsic driver. By integrating segment compatibility constraints (HA/NA) with host ecology, we highlight the PB2 mutational load as a predictive marker for zoonotic potential in contemporary H5Nx lineages.

Our results demonstrate a clear hierarchy of replication efficiency among recombinant PR8 viruses harboring PB2 variants, with MVVTTT_588_ exhibiting the highest competitive fitness (Table 1; Figure 1). The negative impact of the E627K mutation on viral replication in ECEs is well documented, but the lower competitive fitness of MVVTT_339_ and rMVVT_147_ in ECEs was unexpected, given their emergence and frequent detection in circulating wild birds and poultry viruses (Figure 6, see Additional file 2) [13, 40]. Additionally, K339T has been reported to increase polymerase activity in avian cells while reducing replication and virulence in mammals [41]. This paradox may be attributed to segment mismatches, as they appear suboptimal when coupled to the PR8 surface glycoproteins. Replacing PR8 HA and NA with those from a clade 2.3.2.1c strain (K10) restored replication efficiency in ECEs, underscoring the need for coadaptation between PB2 and its cognate HA/NA genes. Among the potential compatibility determinants, NA stalk length is notable. K10 NA contains a 20-amino-acid stalk deletion, whereas PR8 NA retains a shorter, 14-aa deletion; this difference can alter NA catalytic efficiency and the HA–NA functional balance, thereby modulating the performance of specific PB2 variants [42]. Collectively, these findings emphasize that robust viral replication in eggs requires finely tuned interactions among PB2, HA, and NA. Consequently, polymerase mutations must be evaluated within the precise genomic background in which they will be deployed.

The sequence analysis of clade 2.3.2 viruses further corroborated these findings, where we observed parallel changes in HA and PB2 (Table 4). As I147T, K339T, and A588T accumulated after 2003, clade 2.3.2 viruses diversified into subclades 2.3.2.1a, 2.3.2.1b, and 2.3.2.1c in domestic birds in Southeast Asia from 2003 to 2009 [43]. During this evolution, 2.3.2.1c acquired an N-linked glycosylation site (NGS) near a key antigenic region of HA (residue 144, H3 numbering), which is likely to evade vaccine antibodies [25, 44]. The added glycan reduced replication, leading to compensatory V223I mutation to partially restore replication efficiency [25, 26, 44]. The accumulation pattern of these mutations indicates that they initially emerged independently in early clade 2.3.2 viruses but subsequently merged in descendant subclades (2.3.2.1a, b, c). Among the subclades, viruses harboring MVVTTT_588_ mutations eventually dominated in prevalence, particularly within clade 2.3.2.1c, suggesting superior HA–PB2 gene compatibility. Overall, sustained detection of 144NG, V223I, and MVVTTT_588_ mutations highlights the coevolutionary dynamics critical for evolutionary success in vaccinated poultry hosts.

Despite its poultry origin, MVVT_339_ was prevalent among human hosts between 2003 and 2014 (see Additional file 2). However, these viruses are genetically similar to poultry-derived H5N1 isolates, suggesting that poultry is the original and preferable host of K339T [45]. Because MVVTT_339_ replicates poorly in mammalian cells, its persistence in humans almost certainly depends on the later acquisition of mammal-adaptive changes, most notably E627K. Adding E627K (MVVTTK_627_) sharply increases polymerase activity and virus yield in mammalian systems, and inoculation of rMVVTTT_588_ in mice led to rapid emergence of E627K, resulting in fitness comparable to that of rPR8. Although the I147T and I147T–K339T mutations pose little direct pathogenic risk to mammals, their incremental accumulation during repetitive poultry infections may lead to the development of genetic scaffolds in which critical changes such as E627K can arise, increasing zoonotic risk. Mechanistically, E627K increase the interaction of PB2 with the human-preferred host factor ANP32B, which is an interaction that swine, canine, and equine isoforms lack [46]. Humans therefore may act as an amplifying host of E627K. The mutation appears most frequently in human isolates during the evolutionary step from MVV to MVVT_147_, MVVTT_339_, and MVVTTT_588_, and its prevalence is greater in domestic birds, which frequently come into contact with humans, than in wild birds (Figure 5, see Additional file 1). The natural recovery of E627K-positive HPAIVs from poultry supports a scenario in which viruses that acquire E627K in humans are reintroduced to flocks by reverse zoonosis and, facing little competition, can be maintained before spilling over again [12].

Despite the overall dominance of E627K-positive viruses in humans, MVVTTK_627_ was higher among both wild and domestic birds from 2003 to 2006, which coincided with the Qinghai Lake-origin H5N1 outbreaks (Figure 5) [47–49]. Because E627K incurs lower costs in wild birds than in poultry because of differences in avian ANP32A isoforms, spontaneous acquisition of E627K in avian hosts remains improbable [48, 50]. Instead, wild mammals in shared ecosystems likely act as intermediate hosts, allowing the mutation to arise under mammalian selection and then spill back into both migratory and domestic birds, contributing to the Qinghai Lake-origin H5N1 outbreaks [51–53].

Recombinant viruses carrying MVVTTT_588_ presented superior replicative fitness in ECEs regardless of subtypes and moderately enhanced replication efficiency in mammalian cells, which is consistent with a previous study reporting that the same triad increases the virulence and replication potential of low-pathogenicity influenza strains in mice [14]. This ambivalent fitness advantage reflects the dual host adaptation of I147T and A588T (Figure 6, see Additional file 2). After the I147T mutation entered the swine population in the late 1980s, when reassortment between avian influenza viruses and human seasonal viruses occurred in swine herds, it rapidly became widespread in swine hosts [54]. A588T first arose in birds, but MVVT_147_T_588_ first emerged and subsequently became highly prevalent in American swine viruses, indicating adaptive selection within swine hosts [55]. Critically, MVVT_147_T_588_ combined with G590S and Q591R formed the genetic scaffold that gave rise to the swine-origin H1N1 virus responsible for the 2009 pandemic [13, 56, 57]. Thus, the repeated emergence and stable maintenance of these mutations highlight their broad adaptive relevance to both avian and swine hosts, with minimal fitness costs.

No swine isolates have been found with MVVTT_339_ or MVVTTT_588_, underscoring the poultry-specific adaptation conferred by K339T. After accumulation of MVVTT_339_ in avian hosts, A588T accumulation likely occurred independently in avian hosts, whose prevalence rose sharply from 2007, especially within clade 2.3.2.1c viruses (Figure 9) [13, 14]. MVVTTT_588_ peaked between 2009 and 2015 in both avian and human isolates, the same period during which clade 2.3.2.1c H5N1 viruses dominated global outbreaks [43]. Although detections were scarce from 2016 to 2018, the mutation reemerged in a human H5N1 case in the Laos Democratic Republic (LDR) in 2020 and in the fatal Cambodian father-and-daughter case in 2023, highlighting continued zoonotic risk [58–60]. We previously showed that the PR8-based K10 virus replicated in mouse lungs without causing weight loss [26, 39]. Although the polybasic HA cleavage site is not essential for inducing severe weight loss in mice, as previous reports of the recombinant H9N2 virus suggested, it remains an important virulence determinant that is likely to increase pathogenicity in humans [38]. The fact that severe or fatal H5N1 infections still occur mainly in children, who lack the cross-protective immunity present in most adults, further highlights concern [61]. Collectively, these observations suggest that the combination of MVVTTT_588_ in PB2 and polybasic HA cleavage sites likely elevates zoonotic and pathogenic risk in humans, warranting close surveillance and cautious evaluation.

Mass vaccination programs are well-documented drivers of influenza virus evolution [26, 62]. China’s recommended national vaccine against H5Nx HPAIVs, administered since 2003, has been continuously reformulated in response to circulating viral clades [35–37]. Geographical and temporal patterns in the spread of MVVTTT_588_ mutations across multiple H5Nx clades underscore its importance for viral replication in poultry populations, even under intensive vaccination programs. Importantly, gaps in vaccination coverage correlated closely with the reemergence and persistence of MVVTTT_588_-positive viruses. The initial disappearance of MVVTTT_588_ in clade 2.3.4 viruses after 2008 coincided with clade-specific vaccination efforts in China [35, 36]. Similarly, extensive vaccination against clade 2.3.2 viruses starting in 2012 correlated with a decrease in the frequency of MVVTTT_588_-positive 2.3.2.1a and b viruses after 2014. However, the mutation persisted within clade 2.3.2.1c viruses, predominantly in Vietnam, until vaccines targeting clade 2.3.2.1d were reported to be introduced in the region [36]. Notably, adjacent countries such as Cambodia and Laos that did not adopt mass vaccination strategies despite the continuous occurrence of HPAIVs continued to report MVVTTT_588_-carrying clade 2.3.2.1c viruses after 2015, underscoring the role of vaccination strategies in shaping regional virus evolution and persistence [63, 64]. Moreover, the resurgence of MVVTTT_588_ in clade 2.3.4.4 viruses in China since 2013 coincided with the acquisition of internal genes from clade 2.3.2.1c viruses and an interruption in vaccination coverage between 2012 and 2016 [65, 66]. Recent human infections involving MVVTTT_588_-positive viruses, especially in Southeast Asia, highlight the need for sustained genomic surveillance and adaptive vaccination strategies to address antigenic mismatch and continuous viral evolution. Causality cannot be inferred from surveillance-based, policy-level data alone, as our vaccination variable reflects reported/officially recommended clades rather than realized coverage. Although heterogeneity in coverage and reporting lags should be considered, the temporal associations between vaccine-clade shifts and PB2-triad trajectories are consistent with population-level selection, underscoring effective, continuous vaccination as a key public health strategy to mitigate risks posed by highly adaptive influenza viruses.

Clade 2.3.4 followed a trajectory similar to that of clade 2.3.2.1c but began several years earlier. The acquisition of 158-NGS likely compensated for the reduced receptor binding, echoing the 144-NGS in clade 2.3.2.1c. The YN/98 outlier, which shows NA-stalk truncation without HA glycosylation, suggests that stalk deletion can precede HA changes, challenging the conventional sequence of events that regard NA stalk deletion as a subsequent mutation that balances decreased HA activity during the adaptation of wild-bird-derived viruses to poultry [19]. The strong immunogenicity of NA has been reported in various studies, and because a shortened stalk diminishes NA surface exposure and antigenicity, long-term H5N1 vaccination since 2004 appears to favor viruses with truncated N1 [67–69]. Consistent with this, many poultry-derived clade 2.3.2 H5N1 viruses also bear a short NA stalk without HA mutations, as does N270D in NA, which was previously identified as an immune escape mutation (see Additional files 6 and 7) [18]. Interestingly, some clade 2.3.4 H5N1 viruses incorporated the mutated N1 genome of clade 2.3.2 viruses, with an increasing prevalence of N270D mutations over time (see Additional files 7 and 8). Clade 2.3.4 also repeatedly replaced N1 with NA genes from other subtypes, as evidenced by A/chicken/Tibet/LZ01/2010, which possessed a clade 2.3.4 H5N1-like HA (98.4% amino acid identity, with 158-NGS), MVVTT_339_ PB2, and a H9N2-like N2 (A/chicken/Shandong/02/2008; 98.7% amino acid identity), underscoring that anti-N1 antibody pressure can drive extensive NA diversification and even subtype switching (see Additional file 9). As clade 2.3.4 H5Nx continued to diversify, clade 2.3.4.4 H5Nx viruses without 158-NGS and lacking MVVTT_339_ or MVVTTT_588_ emerged. These strains may have been introduced from migratory wild birds, potentially introducing new NA subtypes into poultry populations (see Additional file 10). Collectively, these observations imply that vaccination may promote NA subtype shifts and that NA subtype matching needs to be considered for optimal vaccine development and programs.

In summary, the mutation triad MVVTTT_588_ confers balanced fitness that supports replication in both avian and mammalian hosts, thereby creating a permissive platform for subsequent high-risk adaptations such as E627K and increasing the zoonotic potential of H5Nx viruses. Furthermore, the interdependent relationship among these three mutations in H5N1 HPAIVs underscores the need to consider PB2 evolution together with coordinated changes in HA and NA. Even so, sustained vaccination remains the most effective brake on viral adaptation, limiting diversification and lowering the probability of cross-species spillover.

## Supplementary Information

**Additional file 1.** **Comparison of the E627K mutation frequency among H5Nx HPAIVs from humans and birds.**

**Additional file 2.** **The emergence of mutations in avian, swine, and human hosts.** Mutation frequency and number of PB2 sequences in avian (wild bird and poultry, blue), swine (red), and human (green) viruses harboring MVV-I147T (A), MVV-K339T (B), and MVV-A588T (C). The sequence names depicted in bold letters indicate the first isolated virus in each host. All the analyzed sequences were collected from the GISAID database on 2023.09.12, and human H1 and H3 subtypes were excluded from the analysis.

**Additional file 3.** **Sequence comparison of PB2 variants containing MVVTTT588 from clade 2.3.4 and 2.3.2.1 viruses isolated from 2006-2007.**

**Additional file 4.** **Frequency of MVVT147, MVVTT339, and MVVTTT588 mutations in the PB2 sequences of H5Nx viruses isolated from 2005 to 2014.**

**Additional file 5.** **Sequence analysis of clade 2.3.4 H5N1 viruses isolated from 2005 to 2006.**

**Additional file 6.** **Sequence analysis of clade 2.3.2 viruses isolated from 2004 to 2005.**

**Additional file 7.** **Frequency of N1 mutations among clade 2.3.2 and clade 2.3.4 H5N1 viruses.**

**Additional file 8.** **Sequence comparison of HA and NA genes from clade 2.3.4 viruses isolated in 2005.**

**Additional file 9.** **Sequence comparison of HA and NA genes from clade 2.3.4 viruses isolated in 2010.**

**Additional file 10.** **Sequence analysis of clade 2.3.4 and clade 2.3.4.4 H5Nx viruses without epitope-masking mutations.**

## Data Availability

The datasets used and/or analyzed during the current study are available from the corresponding author on reasonable request.
