# Supplementary material for: Evolutionary trajectories and zoonotic potential of a PB2 mutation triad (I147T, K339T, and A588T) in avian influenza viruses
Source: Vet Res. 2025 Dec 8;57:8. doi: 10.1186/s13567-025-01680-z (PMC12797896; doi:10.1186/s13567-025-01680-z)
Supplement: Supplementary file 1 — Additional file 1. Comparison of the E627K mutation frequency among H5Nx HPAIVs from humans and birds. [file 13567_2025_1680_MOESM1_ESM.docx]

**Additional file 1. Comparison of the E627K mutation frequency among H5Nx HPAIVs from humans and birds.**

| Period  (Vaccine ^a^) | Host ^b^ | # of seq. | Frequency [% (number)] | | | | | | | | | |  |
| --- | --- | --- | --- | --- | --- | --- | --- | --- | --- | --- | --- | --- | --- |
|  |  |  | MVVK_627_ | | MVVTK_627_ | | MVVT_339_K_627_ | | MVVTTK_627_ | | MVVTTTK_627_ | | Total |
| 2003-2006  (N-28, Re1) | H | 151 | **21.19 (32)** | | 0 | | **2.65 (4)** | | 1.99 (3) | | **1.99 (3)** | | **27.81 (42)** |
|  | B | 757 | 1.59 (12) | W^c^: 91.67% (11) | **0.13 (1)** | W: 0 | 0.13 (1) | W: 0 | **16.51 (125)** | W: 40% (50) | 0 | W: 0 | 18.36 (139) |
|  |  |  |  | D^c^: 8.33% (1) |  | D: **100% (1)** |  | D: 100% (1) |  | D: 60% (75) |  | D: 0 |  |
| 2007  (Re1, Re4) | H | 24 | 0 | | 0 | | 0 | | **54.17 (13)** | | 0 | | **54.17 (13)** |
|  | B | 368 | 0.27 (1) | W: 100% (1) | 0 | W: 0 | **0.27 (1)** | W: 0 | 22.28 (82) | W: 15.85% (13) | 0 | W: 0 | 22.83 (84) |
|  |  |  |  | D: 0 |  | D: 0 |  | **D: 100% (1)** |  | D: 84.15% (69) |  | D: 0 |  |
| 2008  (Re-1,  Re-4, Re-5) | H | 28 | 0 | | **3.57 (1)** | | **3.57 (1)** | | **28.57 (8)** | | 0 | | **35.71 (10)** |
|  | B | 208 | 0 | W: 0 | 0 | W: 0 | 0.96 (2) | W: 0 | 18.27 (38) | W: 2.63% (1) | 0 | W: 0 | 19.23 (40) |
|  |  |  |  | D: 0 |  | D: 0 |  | D: 100% (2) |  | D: 97.37% (37) |  | D: 0 |  |
| 2009-2011  (Re-4, Re-5) | H | 99 | 3.03 (9) | | **5.05 (5)** | | **1.01 (1)** | | **44.44 (44)** | | 0 | | **53.53 (53)** |
|  | B | 640 | 0.78 (5) | W: 20% (1) | 0 | W: 0 | 0 | W: 0 | 9.38 (60) | W: 0 | 0 | W: 0 | 10.16 (65) |
|  |  |  |  | D: 80% (4) |  | D: 0 |  | D: 0 |  | D: 100% (60) |  | D: 0 |  |
| 2012  (Re-4, Re-5, Re-6) | H | 11 | **9.09 (1)** | | 0 | | **9.09 (1)** | | **9.09 (1)** | | 0 | | **27.27 (3)** |
|  | B | 266 | 0 | W: 0 | 0 | W: 0 | 0 | W: 0 | 2.26 (6) | W: 0 | **0.38 (1)** | W: 0 | 2.63 (7) |
|  |  |  |  | D: 0 |  | D: 0 |  | D: 0 |  | D: 100% (6) |  | D: 100% (1) |  |
| 2013  (Re-4, Re-6) | H | 395 | **82.28 (325)** | | 0 | | 0 | | 0.25 (1) | | 0 | | **82.53 (326)** |
|  | B | 223 | 0 | W: 0 | 0 | W: 0 | 0 | W: 0 | **2.69 (6)** | W: 0 | **0.45 (1)** | W: 0 | 3.14 (7) |
|  |  |  |  | D: 0 |  | D: 0 |  | D: 0 |  | D: 100% (6) |  | D: 100% (1) |  |
| 2014-2015  (Re-6, Re-7) | H | 374 | **58.56 (219)** | | 0 | | 0 | | 0 | | **0.53 (2)** | | **59.09 (221)** |
|  | B | 1,989 | 0.10 (2) | W: 0 | **0.10 (2)** | W: 100% (2) | 0 | W: 0 | 0 | W: 0 | 0 | W: 0 | 0.20 (4) |
|  |  |  |  | D: 100% (2) |  | D: 0 |  | D: 0 |  | D: 0 |  | D: 0 |  |
| 2016  (Re-6, Re-7, Re-8) | H | 149 | **37.58 (56)** | | 0 | | 0 | | 0 | | 0 | | **37.58 (56)** |
|  | B | 921 | 0 | W: 0 | 0 | W: 0 | 0 | W: 0 | 0 | W: 0 | 0 | W: 0 | 0 |
|  |  |  |  | D: 0 |  | D: 0 |  | D: 0 |  | D: 0 |  | D: 0 |  |
| 2017  (Re-6, Re-8) | H | 406 | **38.42 (156)** | | 0 | | 0 | | 0 | | 0 | | **38.42 (156)** |
|  | B | 949 | 0 | W: 0 | 0 | W: 0 | 0 | W: 0 | 0 | W: 0 | 0 | W: 0 | 0 |
|  |  |  |  | D: 0 |  | D: 0 |  | D: 0 |  | D: 0 |  | D: 0 |  |
| 2018  (Re-8) | H | 17 | **23.53 (4)** | | 0 | | 0 | | 0 | |  |  | **23.53 (4)** |
|  | B | 547 | 0 | W: 0 | 0 | W: 0 | 0 | W: 0 | 0 | W: 0 | 0 | W: 0 | 0 |
|  |  |  |  | D: 0 |  | D: 0 |  | D: 0 |  | D: 0 |  | D: 0 |  |
| 2019-2021  (Re-11, Re-12) | H | 91 | **4.4 (4)** | | 0 | | 0 | | 0 | | 0 | | **4.39 (4)** |
|  | B | 3,207 | 0.12 (4) | W: 0 | **0.03 (1)** | W: 0 | 0 | W: 0 | 0 | W: 0 | 0 | W: 0 | 0.16 (5) |
|  |  |  |  | D: 100% (4) |  | **D: 100% (1)** |  | D: 0 |  | D: 0 |  | D: 0 |  |
| 2022-2023  (Re-13, Re-14) | H | 17 | **11.76 (2)** | | 0 | | 0 | | 0 | | 0 | | **11.76 (2)** |
|  | B | 9,655 | 0.50 (49) | W: 44.9% (22) | 0 | W: 0 | 0 | W: 0 | 0 | W: 0 | 0 | W: 0 | 0.51 (49) |
|  |  |  |  | D: 55.1% (27) |  | D: 0 |  | D: 0 |  | D: 0 |  | D: 0 |  |

^a^ Re-1 (clade 0), Re-4 (clade 7.2), Re-5 (clade 2.3.4), Re-6 (clade 2.3.2), Re-7 (clade 7.2), Re-8 (clade 2.3.4.4g), Re-11 (clade 2.3.4.4h), and Re-12 (clade 2.3.2.1d).
^b^ H, human; B, bird.
^c^ W, wild bird; D, domestic bird.
