# Supplementary material for: Evolutionary trajectories and zoonotic potential of a PB2 mutation triad (I147T, K339T, and A588T) in avian influenza viruses
Source: Vet Res. 2025 Dec 8;57:8. doi: 10.1186/s13567-025-01680-z (PMC12797896; doi:10.1186/s13567-025-01680-z)
Supplement: Supplementary file 2 — Additional file 2. The emergence of mutations in avian, swine, and human hosts. Mutation frequency and number of PB2 sequences in avian (wild bird and poultry, blue), swine (red), and human (green) viruses harboring MVV-I147T (A), MVV-K339T (B), and MVV-A588T (C). The sequence names depicted in bold letters indicate the first isolated virus in each host. All the analyzed sequences were collected from the GISAID database on 2023.09.12, and human H1 and H3 subtypes were excluded from the analysis. [file 13567_2025_1680_MOESM2_ESM.docx]

**

**

**Additional file 2**. **The emergence of mutations in avian, swine, and human hosts.** Mutation frequency and number of PB2 sequences in avian (wild bird and poultry, blue), swine (red), and human (green) viruses harboring MVV-I147T (A), MVV-K339T (B), and MVV-A588T (C). The sequence names depicted in bold letters indicate the first isolated virus in each host. All the analyzed sequences were collected from the GISAID database on 2023.09.12, and human H1 and H3 subtypes were excluded from the analysis.
