# Supplementary material for: Evolutionary trajectories and zoonotic potential of a PB2 mutation triad (I147T, K339T, and A588T) in avian influenza viruses
Source: Vet Res. 2025 Dec 8;57:8. doi: 10.1186/s13567-025-01680-z (PMC12797896; doi:10.1186/s13567-025-01680-z)
Supplement: Supplementary file 3 — Additional file 3. Sequence comparison of PB2 variants containing MVVTTT588 from clade 2.3.4 and 2.3.2.1 viruses isolated from 2006-2007. [file 13567_2025_1680_MOESM3_ESM.docx]

**Additional file 3. Sequence comparison of PB2 variants containing MVVTTT_588_ from clade 2.3.4 and 2.3.2.1 viruses isolated from 2006-2007.**

| Clade | Strain | GISAID ID | Sequence identity | |  |
| --- | --- | --- | --- | --- | --- |
|  |  |  | Nucleotide | Amino acid | |
| 2.3.4 | A/chicken/Hong Kong/D-06-0947/2006 | EPI_ISL_64309 |  |  | |
| 2.3.2.1 | A/common buzzard/Hong Kong/9213/2007 | EPI_ISL_25696 | 98.40% | 99.20% | |
|  | A/peregrine falcon/Hong Kong/1143/2007 | EPI_ISL_25680 | 99.20% | 99.70% | |
|  | A/chicken/Hunan/3/2007 | EPI_ISL_63441 | 98.20% | 99.00% | |
|  | A/duck/Hunan/3/2007 | EPI_ISL_63485 | 98.50% | 99.40% | |
|  | A/little egret/Hong Kong/8550/2007 | EPI_ISL_25693 | 98.40% | 99.60% | |
|  | A/little egret/Hong Kong/8863/2007 | EPI_ISL_25694 | 98.60% | 99.40% | |
|  | A/grey heron/Hong Kong/3088/2007 | EPI_ISL_25695 | 98.50% | 99.40% | |
