# Supplementary material for: Evolutionary trajectories and zoonotic potential of a PB2 mutation triad (I147T, K339T, and A588T) in avian influenza viruses
Source: Vet Res. 2025 Dec 8;57:8. doi: 10.1186/s13567-025-01680-z (PMC12797896; doi:10.1186/s13567-025-01680-z)
Supplement: Supplementary file 4 — Additional file 4. Frequency of MVVT147, MVVTT339, and MVVTTT588 mutations in the PB2 sequences of H5Nx viruses isolated from 2005 to 2014. [file 13567_2025_1680_MOESM4_ESM.docx]

**Additional file 4**. **Frequency of MVVT_147_, MVVTT_339_, and MVVTTT_588_ mutations in the PB2 sequences of H5Nx viruses isolated from 2005 to 2014.**

| Mutation | | Clade | | 2005 | | 2006 | | 2007 | | 2008 | | 2009 | | 2010 | | 2011 | | 2012 | | 2013 | | 2014 | |
| --- | --- | --- | --- | --- | --- | --- | --- | --- | --- | --- | --- | --- | --- | --- | --- | --- | --- | --- | --- | --- | --- | --- | --- |
| MVVT_147_ | | 2.3.4 | | * | |  | | 6.82 | | 10.53 | |  | |  | |  | |  | |  | |  | |
|  |  | 2.3.2 | |  | |  | |  | |  | |  | |  | |  | |  | |  | |  | |
|  |  | 2.3.2.1 | |  | |  | |  | |  | |  | |  | |  | |  | |  | |  | |
|  |  | 2.3.2.1a | |  | |  | |  | |  | |  | |  | | 4.08 | |  | | 20.00 | | 18.75 | |
|  |  | 2.3.2.1b | |  | |  | |  | |  | |  | |  | |  | |  | |  | |  | |
|  |  | 2.3.2.1c | |  | |  | |  | |  | |  | | 4.35 | |  | |  | | 6.74 | | 29.73 | |
|  |  | 2.3.4.4 | |  | |  | |  | |  | |  | |  | |  | |  | |  | |  | |
|  |  | 2.3.4.4b | |  | |  | |  | |  | |  | |  | |  | |  | |  | |  | |
| MVVTT_339_ | | 2.3.4 | | 53.33 | | 36.67 | | 79.55 | | 10.53 | |  | | 50.00 | | 66.67 | |  | |  | |  | |
|  |  | 2.3.2 | | 18.18 | |  | |  | |  | |  | |  | |  | |  | |  | |  | |
|  |  | 2.3.2.1 | |  | |  | |  | |  | |  | |  | |  | |  | |  | |  | |
|  |  | 2.3.2.1a | |  | |  | |  | |  | | 33.33 | | 10.53 | | 39.80 | | 0.81 | | 40.00 | | 81.25 | |
|  |  | 2.3.2.1b | |  | |  | |  | |  | |  | |  | |  | |  | |  | |  | |
|  |  | 2.3.2.1c | |  | |  | |  | |  | | 11.76 | |  | |  | |  | |  | |  | |
|  |  | 2.3.4.4 | |  | |  | |  | |  | |  | |  | |  | |  | |  | | 1.01 | |
|  |  | 2.3.4.4b | |  | |  | |  | |  | |  | |  | |  | |  | |  | | 1.41 | |
| MVVTTT_588_ | | 2.3.4 | |  | | 3.33 | | 2.27 | |  | |  | |  | |  | |  | |  | |  | |
|  | 2.3.2 | |  | |  | |  | |  | |  | |  | |  | |  | |  | |  | |  |
|  | 2.3.2.1 | |  | |  | | 100 | | 94.44 | |  | |  | |  | |  | |  | |  | |  |
|  | 2.3.2.1a | |  | |  | |  | |  | | 66.67 | | 89.47 | | 53.06 | | 45.97 | | 30.00 | |  | |  |
|  | 2.3.2.1b | |  | |  | |  | |  | | 100 | | 100 | | 90.32 | | 100 | | 100 | |  | |  |
|  | 2.3.2.1c | |  | |  | |  | |  | | 88.24 | | 91.30 | | 93.94 | | 91.38 | | 74.16 | | 60.81 | |  |
|  | 2.3.4.4 | |  | |  | |  | |  | |  | |  | |  | |  | | 80.95 | | 82.83 | |  |
|  | 2.3.4.4b | |  | |  | |  | |  | |  | |  | |  | |  | | 44.44 | | 50.70 | |  |

*Shaded blocks indicate no isolates
