# Supplementary material for: Evolutionary trajectories and zoonotic potential of a PB2 mutation triad (I147T, K339T, and A588T) in avian influenza viruses
Source: Vet Res. 2025 Dec 8;57:8. doi: 10.1186/s13567-025-01680-z (PMC12797896; doi:10.1186/s13567-025-01680-z)
Supplement: Supplementary file 5 — Additional file 5. Sequence analysis of clade 2.3.4 H5N1 viruses isolated from 2005 to 2006. [file 13567_2025_1680_MOESM5_ESM.docx]

**Additional file 5. Sequence analysis of clade 2.3.4 H5N1 viruses isolated from 2005 to 2006**

| Strain | Subtype | GISAID ID | Isolated year | HA residues | | NA residues |  |
| --- | --- | --- | --- | --- | --- | --- | --- |
|  |  |  |  | 144-146* | 158-160 | stalk deletion | |
| A/turtle_dove/Guangdong/300/2005 | H5N1 | EPI_ISL_222486 | 2005 | MPS | NNT | 20 amino acid deletion | |
| A/duck/Hunan/316/2005 | H5N1 | EPI_ISL_181276 | 2005 | TPS | NNT | 20 amino acid deletion | |
| A/chicken/Ninh_Binh/209/2005 | H5N1 | EPI_ISL_64835 | 2005 | TPS | NNT | 20 amino acid deletion | |
| A/Mallard/Huadong/S/2005 | H5N1 | EPI_ISL_64892 | 2005 | TPS | NNT | 20 amino acid deletion | |
| A/Mallard/Huadong/lk/2005 | H5N1 | EPI_ISL_64894 | 2005 | TPS | NNT | 20 amino acid deletion | |
| A/wild_duck/Hunan/021/2005 | H5N1 | EPI_ISL_13988 | 2005 | TPS | NNT | 20 amino acid deletion | |
| A/chicken/Hunan/21/2005 | H5N1 | EPI_ISL_77988 | 2005 | TPS | NNT | 20 amino acid deletion | |
| A/wild_duck/Hunan/211/2005 | H5N1 | EPI_ISL_13989 | 2005 | TPS | NNT | 20 amino acid deletion | |
| A/chicken/Sichuan/81/2005 | H5N1 | EPI_ISL_78000 | 2005 | TPS | NNT | 20 amino acid deletion | |
| A/duck/Anhui/56/2005 | H5N1 | EPI_ISL_78012 | 2005 | TPS | NNT | 20 amino acid deletion | |
| A/duck/Hubei/49/2005 | H5N1 | EPI_ISL_78014 | 2005 | TPS | NNT | 20 amino acid deletion | |
| A/duck/Jiangxi/80/2005 | H5N1 | EPI_ISL_78019 | 2005 | TPS | NNT | 20 amino acid deletion | |
| A/duck/Vietnam/219/2005 | H5N1 | EPI_ISL_27331 | 2005 | TPS | NNT | 20 amino acid deletion | |
| A/duck/Vietnam/218/2005 | H5N1 | EPI_ISL_27332 | 2005 | TPS | NNT | 20 amino acid deletion | |
| A/Muscovy_duck/Vietnam/217/2005 | H5N1 | EPI_ISL_27333 | 2005 | TPS | NNT | 20 amino acid deletion | |
| A/chicken/Vietnam/216/2005 | H5N1 | EPI_ISL_27334 | 2005 | TPS | NNT | 20 amino acid deletion | |
| A/goose/Hubei/65/2005 | H5N1 | EPI_ISL_78022 | 2005 | TPS | NNT | 20 amino acid deletion | |
| A/duck/Vietnam/215/2005 | H5N1 | EPI_ISL_27335 | 2005 | TPS | NNT | 20 amino acid deletion | |
| A/Muscovy_duck/Vietnam/213/2005 | H5N1 | EPI_ISL_27336 | 2005 | TPS | NNT | 20 amino acid deletion | |
| A/chicken/Vietnam/212/2005 | H5N1 | EPI_ISL_27337 | 2005 | TPS | NNT | 20 amino acid deletion | |
| A/Muscovy_duck/Vietnam/211/2005 | H5N1 | EPI_ISL_27338 | 2005 | TPS | NNT | 20 amino acid deletion | |
| A/duck/Vietnam/210/2005 | H5N1 | EPI_ISL_27339 | 2005 | TPS | NNT | 20 amino acid deletion | |
| A/chicken/Vietnam/209/2005 | H5N1 | EPI_ISL_27340 | 2005 | TPS | NNT | 20 amino acid deletion | |
| A/duck/Vietnam/208/2005 | H5N1 | EPI_ISL_27341 | 2005 | TPS | NNT | 20 amino acid deletion | |
| A/duck/Vietnam/207/2005 | H5N1 | EPI_ISL_27342 | 2005 | TPS | NNT | 20 amino acid deletion | |
| A/duck/Vietnam/205/2005 | H5N1 | EPI_ISL_27344 | 2005 | TPS | NNT | 20 amino acid deletion | |
| A/chicken/Vietnam/202/2005 | H5N1 | EPI_ISL_27347 | 2005 | TPS | NNT | 20 amino acid deletion | |
| A/chicken/Vietnam/200/2005 | H5N1 | EPI_ISL_27349 | 2005 | TSS | NNS | 20 amino acid deletion | |
| A/chicken/Hunan/3157/2006 | H5N1 | EPI_ISL_29187 | 2006 | TPS | NNT | 20 amino acid deletion | |
| A/duck/Hunan/3315/2006 | H5N1 | EPI_ISL_29188 | 2006 | TPS | NNT | 20 amino acid deletion | |
| A/duck/Hunan/3340/2006 | H5N1 | EPI_ISL_29189 | 2006 | TPS | NNT | 20 amino acid deletion | |
| A/duck/Yunnan/4873/2006 | H5N1 | EPI_ISL_24610 | 2006 | TPS | NNT | 20 amino acid deletion | |
| A/duck/Yunnan/5310/2006 | H5N1 | EPI_ISL_24611 | 2006 | TPS | NNT | 20 amino acid deletion | |
| A/goose/Yunnan/3798/2006 | **H5N1** | **EPI_ISL_24613** | **2006** | **MPS** | **NNI** | **20 amino acid deletion** | |
| A/goose/Yunnan/4371/2006 | H5N1 | EPI_ISL_24614 | 2006 | TPS | NNT | 20 amino acid deletion | |
| A/goose/Yunnan/4389/2006 | H5N1 | EPI_ISL_24615 | 2006 | TPS | NNT | 20 amino acid deletion | |
| A/goose/Yunnan/4985/2006 | H5N1 | EPI_ISL_24616 | 2006 | TPS | NNT | 20 amino acid deletion | |
| A/goose/Yunnan/5769/2006 | H5N1 | EPI_ISL_24620 | 2006 | TPS | NNT | 20 amino acid deletion | |
| A/goose/Yunnan/5979/2006 | H5N1 | EPI_ISL_24621 | 2006 | TPS | NNT | 20 amino acid deletion | |
| A/chicken/Hong_Kong/D-06-0947/2006 | H5N1 | EPI_ISL_64309 | 2006 | TPS | NNT | 20 amino acid deletion | |
| A/duck/Laos/25/2006 | H5N1 | EPI_ISL_30555 | 2006 | TPS | NNT | 20 amino acid deletion | |
| A/duck/Hai_Phong_/208/2006 | H5N1 | EPI_ISL_64352 | 2006 | TPS | NNT | 20 amino acid deletion | |
| A/Chicken/Laos/Xaythiani_26/2006 | H5N1 | EPI_ISL_109927 | 2006 | TPS | NNT | 20 amino acid deletion | |
| A/domestic_green-winged_teal/Hunan/3450/2006 | H5N1 | EPI_ISL_138602 | 2006 | TPS | NNT | 20 amino acid deletion | |
| A/duck/Anhui/1/06 | H5N1 | EPI_ISL_78011 | 2006 | KPS | NNT | 20 amino acid deletion | |
| A/duck/Hubei/Hangmei01/2006 | H5N1 | EPI_ISL_29372 | 2006 | TPS | NNT | 20 amino acid deletion | |
| A/duck/Hunan/29/2006 | H5N1 | EPI_ISL_78016 | 2006 | MPS | NNT | 20 amino acid deletion | |
| A/duck/Yunnan/47/2006 | H5N1 | EPI_ISL_78020 | 2006 | MPS | NNT | 20 amino acid deletion | |
| A/shrike/Tibet/13/2006 | H5N1 | EPI_ISL_78025 | 2006 | TPS | NNT | 20 amino acid deletion | |
| A/duck/Hunan/689/2006 | H5N1 | EPI_ISL_29174 | 2006 | TPS | NNT | 20 amino acid deletion | |
| A/chicken/Henan/1362/2006 | H5N1 | EPI_ISL_29176 | 2006 | TPS | NNT | 20 amino acid deletion | |
| A/duck/Henan/1650/2006 | H5N1 | EPI_ISL_29178 | 2006 | TPS | NNT | 20 amino acid deletion | |

*H3 numbering
