# Supplementary material for: Evolutionary trajectories and zoonotic potential of a PB2 mutation triad (I147T, K339T, and A588T) in avian influenza viruses
Source: Vet Res. 2025 Dec 8;57:8. doi: 10.1186/s13567-025-01680-z (PMC12797896; doi:10.1186/s13567-025-01680-z)
Supplement: Supplementary file 6 — Additional file 6. Sequence analysis of clade 2.3.2 viruses isolated from 2004 to 2005. [file 13567_2025_1680_MOESM6_ESM.docx]

**Additional file 6. Sequence analysis of clade 2.3.2 viruses isolated from 2004 to 2005**

| Strain | subtype | GISAID ID | isolated year | HA |  | NA |
| --- | --- | --- | --- | --- | --- | --- |
|  |  |  |  | 144-146* | 158-160 | stalk deletion |
| A/goose/Yunnan/4129/2005 | H5N1 | EPI_ISL_10752 | 2005 | SSS | NNT | 20 amino acid deletion |
| A/duck/Yunnan/4400/2005** | **H5N1** | **EPI_ISL_10753** | **2005** | **SSS** | **NNA** | **20 amino acid deletion** |
| A/goose/Yunnan/4494/2005 | **H5N1** | **EPI_ISL_10754** | **2005** | **SSS** | **NNA** | **20 amino acid deletion** |
| A/duck/Yunnan/4589/2005 | H5N1 | EPI_ISL_10755 | 2005 | SSS | NNT | 20 amino acid deletion |
| A/goose/Yunnan/4804/2005 | H5N1 | EPI_ISL_10756 | 2005 | SSS | NNT | 20 amino acid deletion |
| A/duck/Yunnan/5877/2005 | **H5N1** | **EPI_ISL_10757** | **2005** | **SSS** | **NNA** | **20 amino acid deletion** |
| A/goose/Yunnan/6027/2005 | **H5N1** | **EPI_ISL_10758** | **2005** | **SSS** | **NNA** | **20 amino acid deletion** |
| A/duck/Yunnan/6332/2005 | H5N1 | EPI_ISL_10759 | 2005 | SSS | NNT | 20 amino acid deletion |
| A/quail/Viet_Nam/15/2005 | H5N1 | EPI_ISL_6415 | 2005 | NSS | NDA | 20 amino acid deletion |
| A/chicken/Viet_Nam/17/2005 | H5N1 | EPI_ISL_6416 | 2005 | NSS | NDA | 20 amino acid deletion |
| A/duck/China/E319-2/03 | **H5N1** | **EPI_ISL_3740** | **2003** | **SSS** | **NDA** | **20 amino acid deletion** |
| A/silky_chicken/Shantou/475/2004 | **H5N1** | **EPI_ISL_15646** | **2004** | **SSS** | **NNA** | **20 amino acid deletion** |
| A/chicken/Vietnam/TY31/2005 | H5N1 | EPI_ISL_13218 | 2005 | NSS | NDA | 20 amino acid deletion |
| A/duck/Guangxi/351/2004 | **H5N1** | **EPI_ISL_9896** | **2004** | **SSS** | **NNA** | **20 amino acid deletion** |
| A/duck/Guangxi/380/2004 | **H5N1** | **EPI_ISL_9897** | **2004** | **SSS** | **NNA** | **20 amino acid deletion** |
| A/chicken/Vietnam/TY25/2005 | H5N1 | EPI_ISL_13226 | 2005 | NSS | NDA | 20 amino acid deletion |
| A/chicken/Guiyang/2147/2005 | H5N1 | EPI_ISL_10670 | 2005 | SSS | NNT | 20 amino acid deletion |
| A/chicken/Guiyang/2173/2005 | H5N1 | EPI_ISL_10671 | 2005 | SSS | NNT | 20 amino acid deletion |
| A/duck/Yunnan/5251/2005 | H5N1 | EPI_ISL_10673 | 2005 | SSS | NNT | 20 amino acid deletion |
| A/duck/Yunnan/5820/2005 | H5N1 | EPI_ISL_10674 | 2005 | SSS | NNT | 20 amino acid deletion |
| A/goose/Yunnan/6368/2005 | H5N1 | EPI_ISL_10675 | 2005 | SSS | NNT | 20 amino acid deletion |
| A/duck/Yunnan/6607/2005 | H5N1 | EPI_ISL_10676 | 2005 | SSS | NNT | 20 amino acid deletion |
| A/chicken/Guangdong/178/04 | **H5N1** | **EPI_ISL_4532** | **2004** | **SSS** | **NNA** | **20 amino acid deletion** |
| A/chicken/Guangxi/2448/2004 | H5N1 | EPI_ISL_9913 | 2004 | SSS | NNA | No deletion |
| A/chicken/Guangxi/2461/2004 | **H5N1** | **EPI_ISL_9914** | **2004** | **SSS** | **NNA** | **20 amino acid deletion** |
| A/goose/Guangxi/345/2005 | **H5N1** | **EPI_ISL_9915** | **2005** | **SSS** | **NNA** | **20 amino acid deletion** |
| A/duck/Guangdong/23/2004 | **H5N1** | **EPI_ISL_78013** | **2004** | **SSS** | **NNA** | **20 amino acid deletion** |
| A/quail/Guangxi/575/2005 | **H5N1** | **EPI_ISL_9916** | **2005** | **SSS** | **DNA** | **20 amino acid deletion** |
| A/chicken/Guangxi/604/2005 | **H5N1** | **EPI_ISL_9917** | **2005** | **SSS** | **DNA** | **20 amino acid deletion** |
| A/duck/Guangxi/793/2005 | **H5N1** | **EPI_ISL_9918** | **2005** | **SSS** | **DNA** | **20 amino acid deletion** |
| A/duck/Guangxi/951/2005 | **H5N1** | **EPI_ISL_9919** | **2005** | **SSS** | **NNA** | **20 amino acid deletion** |
| A/chicken/Guangdong/1/2005 | **H5N1** | **EPI_ISL_23104** | **2005** | **SSS** | **NNA** | **20 amino acid deletion** |
| A/chicken/Lang_Son/200/2005 | H5N1 | EPI_ISL_64832 | 2005 | SSS | NNS | 20 amino acid deletion |
| A/duck/Hunan/1265/2005 | **H5N1** | **EPI_ISL_9930** | **2005** | **SSS** | **NNA** | **20 amino acid deletion** |
| A/duck/Hunan/1608/2005 | **H5N1** | **EPI_ISL_9931** | **2005** | **SSS** | **NNA** | **20 amino acid deletion** |
| A/duck/Hunan/1652/2005 | **H5N1** | **EPI_ISL_9932** | **2005** | **SSS** | **NNA** | **20 amino acid deletion** |
| A/duck/Vietnam/206/2005 | H5N1 | EPI_ISL_27343 | 2005 | SSS | NNS | 20 amino acid deletion |
| A/duck/Vietnam/204/2005 | H5N1 | EPI_ISL_27345 | 2005 | SSS | NNS | 20 amino acid deletion |
| A/duck/Vietnam/203/2005 | H5N1 | EPI_ISL_27346 | 2005 | SSS | NNS | 20 amino acid deletion |
| A/duck/Vietnam/201/2005 | H5N1 | EPI_ISL_27348 | 2005 | SSS | NNS | 20 amino acid deletion |
| A/grey_heron/Hong_Kong/728/2004 | H5N1 | EPI_ISL_9942 | 2004 | SSS | NNA | No deletion |
| A/Ph/ST/44/2004 | **H5N1** | **EPI_ISL_4188** | **2004** | **SSS** | **NNA** | **20 amino acid deletion** |
| A/chicken/Yunnan/chuxiong01/2005 | H5N1 | EPI_ISL_19165 | 2005 | SSS | NNT | 20 amino acid deletion |
| A/duck/Lang_Son/201/2005 | H5N1 | EPI_ISL_64865 | 2005 | SSS | NNS | 20 amino acid deletion |
| A/duck/Vietnam/568/2005 | H5N1 | EPI_ISL_9958 | 2005 | NSS | NDA | 20 amino acid deletion |
| A/grey_heron/Hong_Kong/837/2004 | H5N1 | EPI_ISL_9959 | 2004 | SSS | NNA | No deletion |
| A/Chinese_pond_heron/Hong_Kong/18/2005 | H5N1 | EPI_ISL_9960 | 2005 | SSS | NNA | No deletion |
| A/Chicken/Shantou/810/05 | **H5N1** | **EPI_ISL_9326** | **2005** | **SSS** | **NNA** | **20 amino acid deletion** |
| A/Quail/Shantou/911/05 | **H5N1** | **EPI_ISL_9327** | **2005** | **SSS** | **NNA** | **20 amino acid deletion** |
| A/duck/Guangxi/2926/2005 | **H5N1** | **EPI_ISL_10736** | **2005** | **SSS** | **NNA** | **20 amino acid deletion** |
| A/goose/Guangxi/3017/2005 | **H5N1** | **EPI_ISL_10737** | **2005** | **SSS** | **NNA** | **20 amino acid deletion** |
| A/duck/Guangxi/3085/2005 | **H5N1** | **EPI_ISL_10738** | **2005** | **SSS** | **NNA** | **20 amino acid deletion** |
| A/chicken/Guangxi/3154/2005 | **H5N1** | **EPI_ISL_10739** | **2005** | **SSS** | **NNA** | **20 amino acid deletion** |
| A/goose/Guangxi/3316/2005 | H5N1 | EPI_ISL_10740 | 2005 | NSS | NDA | 20 amino acid deletion |
| A/duck/Guangxi/3364/2005 | **H5N1** | **EPI_ISL_10741** | **2005** | **SSS** | **NNA** | **20 amino acid deletion** |
| A/duck/Guangxi/3548/2005 | H5N1 | EPI_ISL_10742 | 2005 | SSS | NNT | 20 amino acid deletion |
| A/goose/Guangxi/3714/2005 | **H5N1** | **EPI_ISL_10743** | **2005** | **SSS** | **NNA** | **20 amino acid deletion** |
| A/duck/Guangxi/3741/2005 | H5N1 | EPI_ISL_10744 | 2005 | NSS | NNA | 20 amino acid deletion |
| A/chicken/Viet_Nam/10/2005 | **H5N1** | **EPI_ISL_6392** | **2005** | **SSS** | **NNA** | **20 amino acid deletion** |
| A/chicken/Guangxi/3791/2005 | H5N1 | EPI_ISL_10745 | 2005 | NSS | NNA | 20 amino acid deletion |
| A/duck/Guangxi/3819/2005 | H5N1 | EPI_ISL_10746 | 2005 | NSS | NNA | 20 amino acid deletion |
| A/duck/Viet_Nam/12/2005 | H5N1 | EPI_ISL_6394 | 2005 | NSS | NDA | 20 amino acid deletion |
| A/duck/Guangxi/4016/2005 | **H5N1** | **EPI_ISL_10747** | **2005** | **SSS** | **NNA** | **20 amino acid deletion** |
| A/duck/Guangxi/4184/2005 | **H5N1** | **EPI_ISL_10748** | **2005** | **SSS** | **NNA** | **20 amino acid deletion** |
| A/duck/Guangxi/4196/2005 | **H5N1** | **EPI_ISL_10749** | **2005** | **SSS** | **NSA** | **20 amino acid deletion** |
| A/duck/Guangxi/4665/2005 | **H5N1** | **EPI_ISL_10750** | **2005** | **SSS** | **NNA** | **20 amino acid deletion** |
| A/goose/Yunnan/3720/2005 | **H5N1** | **EPI_ISL_10751** | **2005** | **SSS** | **NNA** | **20 amino acid deletion** |

*H3 numbering

**Strains with short NA stalk (20 amino acid deletion) and without HA mutations that mask epitopes are highlighted in bold letters
