# Supplementary material for: Evolutionary trajectories and zoonotic potential of a PB2 mutation triad (I147T, K339T, and A588T) in avian influenza viruses
Source: Vet Res. 2025 Dec 8;57:8. doi: 10.1186/s13567-025-01680-z (PMC12797896; doi:10.1186/s13567-025-01680-z)
Supplement: Supplementary file 7 — Additional file 7. Frequency of N1 mutations among clade 2.3.2 and clade 2.3.4 H5N1 viruses. [file 13567_2025_1680_MOESM7_ESM.docx]

**Additional file 7. Frequency of N1 mutations among clade 2.3.2 and clade 2.3.4 H5N1 viruses.**

| Mutation | 2.3.2  (~2005) | 2.3.4  (~2005) | 2.3.4  (2005~2007) |
| --- | --- | --- | --- |
| H44N | 50.70 | 1.30 | **24.74** |
| A46V | 77.46 | 6.49 | **36.32** |
| I211M | 56.34 | 5.19 | **40.00** |
| **N270D** | **92.96** | **7.79** | **40.00** |
| G382E | 87.32 | 24.68 | **47.37** |
