# Supplementary material for: Evolutionary trajectories and zoonotic potential of a PB2 mutation triad (I147T, K339T, and A588T) in avian influenza viruses
Source: Vet Res. 2025 Dec 8;57:8. doi: 10.1186/s13567-025-01680-z (PMC12797896; doi:10.1186/s13567-025-01680-z)
Supplement: Supplementary file 8 — Additional file 8. Sequence comparison of HA and NA genes from clade 2.3.4 viruses isolated in 2005. [file 13567_2025_1680_MOESM8_ESM.docx]

**Additional file 8. Sequence comparison of HA and NA genes from clade 2.3.4 viruses isolated in 2005.**

|  |  | Clade 2.3.4 viruses with the clade 2.3.2 NA sequence | | | | | |
| --- | --- | --- | --- | --- | --- | --- | --- |
| Clade 2.3.2 H5N1 viruses |  | A/duck/Anhui/56/2005 | | A/chicken/Vietnam/200/2005 | | A/Mallard/Huadong/S/2005 | |
|  | GISAID ID | Nucleotide | Amino acid | Nucleotide | Amino acid | Nucleotide | Amino acid |
| A/goose/Yunnan/4129/2005 | EPI_ISL_10752 | 98.60 | 99.1 | 99.00 | 98.7 | 98.7 | 99.3 |
| A/duck/Yunnan/4400/2005 | EPI_ISL_10753 | 99.00 | 99.3 | 99.30 | 98.9 | 99 | 99.6 |
| A/goose/Yunnan/4494/2005 | EPI_ISL_10754 | 98.90 | 99.3 | 99.30 | 98.9 | 99 | 99.6 |
| A/duck/Yunnan/4589/2005 | EPI_ISL_10755 | 98.60 | 99.1 | 99.00 | 98.7 | 98.7 | 99.3 |
| A/goose/Yunnan/4804/2005 | EPI_ISL_10756 | 99.00 | 99.3 | 99.20 | 98.9 | 99 | 99.6 |
| A/duck/Yunnan/5877/2005 | EPI_ISL_10757 | 98.50 | 99.3 | 98.90 | 98.9 | 98.6 | 99.6 |
| A/duck/Yunnan/6332/2005 | EPI_ISL_10759 | 98.40 | 98.4 | 99.10 | 98.9 | 98.5 | 98.7 |
| A/quail/Viet_Nam/15/2005 | EPI_ISL_6415 | 99.00 | 99.1 | 98.50 | 98.2 | 99.1 | 99.3 |
| A/chicken/Viet_Nam/17/2005 | EPI_ISL_6416 | 99.00 | 99.3 | 98.40 | 98.4 | 99 | 99.6 |
| A/duck/China/E319-2/03 | EPI_ISL_3740 | 98.30 | 97.3 | 97.90 | 97.1 | 98.4 | 97.6 |
| A/silky_chicken/Shantou/475/2004 | EPI_ISL_15646 | 99.00 | 98.9 | 98.50 | 98 | 99.1 | 99.1 |
| A/chicken/Vietnam/TY31/2005 | EPI_ISL_13218 | 99.00 | 99.3 | 98.40 | 98.4 | 99 | 99.6 |
| A/duck/Guangxi/351/2004 | EPI_ISL_9896 | 99.00 | 99.3 | 98.50 | 98.4 | 99.1 | 99.6 |
| A/chicken/Vietnam/TY25/2005 | EPI_ISL_13226 | 99.00 | 98.9 | 98.40 | 98 | 99 | 99.1 |
| A/chicken/Guiyang/2147/2005 | EPI_ISL_10670 | 98.70 | 99.1 | 99.00 | 98.7 | 98.7 | 99.3 |
| A/chicken/Guiyang/2173/2005 | EPI_ISL_10671 | 98.70 | 99.1 | 99.00 | 98.7 | 98.7 | 99.3 |
| A/duck/Yunnan/5251/2005 | EPI_ISL_10673 | 98.60 | 99.3 | 99.00 | 98.9 | 98.7 | 99.6 |
| A/duck/Yunnan/5820/2005 | EPI_ISL_10674 | 98.60 | 99.3 | 99.00 | 98.9 | 98.7 | 99.6 |
| A/goose/Yunnan/6368/2005 | EPI_ISL_10675 | 98.40 | 98.4 | 99.00 | 98.9 | 98.4 | 98.7 |
| A/duck/Yunnan/6607/2005 | EPI_ISL_10676 | 98.40 | 98.4 | 99.00 | 98.9 | 98.4 | 98.7 |
| A/chicken/Guangdong/178/04 | EPI_ISL_4532 | 99.10 | 99.6 | 98.60 | 98.7 | 99.2 | 99.8 |
| A/chicken/Guangxi/2461/2004 | EPI_ISL_9914 | 98.80 | 98.7 | 98.30 | 97.8 | 98.9 | 98.9 |
| A/duck/Guangdong/23/2004 | EPI_ISL_78013 | 98.10 | 97.1 | 97.60 | 96.2 | 98.2 | 97.3 |
| A/quail/Guangxi/575/2005 | EPI_ISL_9916 | 98.90 | 98.7 | 98.20 | 98 | 99 | 98.9 |
| A/chicken/Guangxi/604/2005 | EPI_ISL_9917 | 96.60 | 97.3 | 96.10 | 96.9 | 96.8 | 97.6 |
| A/duck/Guangxi/793/2005 | EPI_ISL_9918 | 96.60 | 97.6 | 96.10 | 97.1 | 96.8 | 97.8 |
| A/duck/Guangxi/951/2005 | EPI_ISL_9919 | 98.90 | 99.1 | 99.10 | 98.7 | 99 | 99.3 |
| A/chicken/Guangdong/1/2005 | EPI_ISL_23104 | 96.10 | 97.1 | 95.80 | 96.9 | 96.3 | 97.3 |
| A/chicken/Lang_Son/200/2005 | EPI_ISL_64832 | 97.30 | 97.8 | 97.30 | 97.8 | 97.3 | 98 |
| A/duck/Hunan/1265/2005 | EPI_ISL_9930 | 99.30 | 99.3 | 98.60 | 98.4 | 99.3 | 99.6 |
| A/duck/Vietnam/206/2005 | EPI_ISL_27343 | 98.30 | 98.2 | 100.00 | 100 | 98.4 | 98.4 |
| A/duck/Vietnam/204/2005 | EPI_ISL_27345 | 98.30 | 98.2 | 100.00 | 100 | 98.4 | 98.4 |
| A/duck/Vietnam/203/2005 | EPI_ISL_27346 | 98.30 | 98.2 | 100.00 | 100 | 98.4 | 98.4 |
| A/duck/Vietnam/201/2005 | EPI_ISL_27348 | 98.30 | 98.2 | 100.00 | 100 | 98.4 | 98.4 |
| A/grey_heron/Hong_Kong/728/2004 | EPI_ISL_9942 | 94.10 | 94.2 | 93.70 | 93.8 | 94.3 | 94.4 |
| A/Ph/ST/44/2004 | EPI_ISL_4188 | 99.30 | 99.6 | 98.80 | 98.7 | 99.4 | 99.8 |
| A/chicken/Yunnan/chuxiong01/2005 | EPI_ISL_19165 | 97.80 | 97.3 | 98.30 | 97.8 | 97.9 | 97.6 |
| A/duck/Lang_Son/201/2005 | EPI_ISL_64865 | 98.20 | 98.2 | 99.90 | 100 | 98.3 | 98.4 |
| A/duck/Vietnam/568/2005 | EPI_ISL_9958 | 98.80 | 99.1 | 98.30 | 98.2 | 98.9 | 99.3 |
| A/grey_heron/Hong_Kong/837/2004 | EPI_ISL_9959 | 94.00 | 93.9 | 93.60 | 93.5 | 94.2 | 94.2 |
| A/Chinese_pond_heron/Hong_Kong/18/2005 | EPI_ISL_9960 | 94.00 | 94.4 | 93.60 | 94 | 94.2 | 94.7 |
| A/Chicken/Shantou/810/05 | EPI_ISL_9326 | 96.10 | 97.1 | 95.80 | 96.9 | 96.3 | 97.3 |
| A/Quail/Shantou/911/05 | EPI_ISL_9327 | 96.40 | 97.6 | 96.10 | 97.3 | 96.6 | 97.8 |
| A/chicken/Viet_Nam/10/2005 | EPI_ISL_6392 | 99.30 | 99.3 | 98.70 | 98.4 | 99.3 | 99.6 |
| A/duck/Viet_Nam/12/2005 | EPI_ISL_6394 | 99.00 | 99.3 | 98.50 | 98.4 | 99.3 | 99.6 |
| A/goose/Yunnan/3720/2005 | EPI_ISL_10751 | 98.90 | 99.3 | 99.30 | 98.9 | 99 | 99.6 |
