# Supplementary material for: Evolutionary trajectories and zoonotic potential of a PB2 mutation triad (I147T, K339T, and A588T) in avian influenza viruses
Source: Vet Res. 2025 Dec 8;57:8. doi: 10.1186/s13567-025-01680-z (PMC12797896; doi:10.1186/s13567-025-01680-z)
Supplement: Supplementary file 9 — Additional file 9. Sequence comparison of HA and NA genes from clade 2.3.4 viruses isolated in 2010. [file 13567_2025_1680_MOESM9_ESM.docx]

**Additional file 9. Sequence comparison of HA and NA genes from clade 2.3.4 viruses isolated in 2010.**

| Strain | Subtype | GISAID ID | NA sequence identity (%) | | HA sequence identity (%) | |
| --- | --- | --- | --- | --- | --- | --- |
|  |  |  | Nucleotide | Amino acid | Nucleotide | Amino acid |
| A/chicken/Tibet/LZ01/2010 | H5N2 | EPI_ISL_129354 | |  |  |  |
| A/chicken/Shandong/02/2008 | H9N2 | EPI_ISL_134189 | 98.3 | 98.7 |  |  |
| A/chicken/Eastern_China/ZG16/2010 | H5N1 | EPI_ISL_151698 |  |  | 98.7 | 98.4 |
| A/chicken/North_China/k0604/2010 | H5N1 | EPI_ISL_151697 |  |  | 98.7 | 98.4 |
