# Supplementary material for: Evolutionary trajectories and zoonotic potential of a PB2 mutation triad (I147T, K339T, and A588T) in avian influenza viruses
Source: Vet Res. 2025 Dec 8;57:8. doi: 10.1186/s13567-025-01680-z (PMC12797896; doi:10.1186/s13567-025-01680-z)
Supplement: Supplementary file 10 — Additional file 10. Sequence analysis of clade 2.3.4 and clade 2.3.4.4 H5Nx viruses without epitope-masking mutations. [file 13567_2025_1680_MOESM10_ESM.docx]

**Additional file 10.** **Sequence analysis of clade 2.3.4 and clade 2.3.4.4 H5Nx viruses without epitope-masking mutations.**

| Strain | subtype | clade | GISAID ID | isolated year | HA residues | | PB2 residues | | | | | |
| --- | --- | --- | --- | --- | --- | --- | --- | --- | --- | --- | --- | --- |
|  |  |  |  |  | 144-146* | 158-160 | 66 | 109 | 133 | 147 | 339 | 588 |
| A/goose/Yunnan/3798/2006 | H5N1 | 2.3.4 | EPI_ISL_24613 | 2006 | MPS | NNI | M | V | V | T | T | A |
| A/duck/Hubei/2911/2007 | H5N1 | 2.3.4 | EPI_ISL_29185 | 2007 | TPS | NNA | M | V | V | I | K | A |
| A/duck/Guangdong/wy19/2008 | H5N5 | 2.3.4 | EPI_ISL_152086 | 2008 | TPS | NDA | M | V | V | I | R | A |
| A/duck/Guangdong/wy24/2008 | H5N5 | 2.3.4 | EPI_ISL_152087 | 2008 | TPS | NNA | M | V | V | I | R | A |
| A/duck/Guangdong/wy11/2008 | H5N5 | 2.3.4 | EPI_ISL_152085 | 2008 | TPS | NDA | M | V | V | I | R | A |
| A/duck/Eastern_China/008/2008 | H5N5 | 2.3.4 | EPI_ISL_94329 | 2008 | TPS | NNA | M | V | V | I | R | A |
| A/goose/Shandong/k1204/2009 | H5N5 | 2.3.4 | EPI_ISL_139383 | 2009 | TPS | NNA | V | V | V | I | R | A |
| A/duck/Eastern_China/031/2009 | H5N5 | 2.3.4 | EPI_ISL_94330 | 2009 | TPS | NNA | M | V | V | I | R | A |
| A/quail/Jiangsu/k0104/2010 | H5N5 | 2.3.4 | EPI_ISL_139386 | 2010 | TPS | NNA | M | V | V | I | K | A |
| A/goose/Guangdong/k0103/2010 | H5N5 | 2.3.4 | EPI_ISL_139384 | 2010 | TPS | NNA | V | V | V | I | R | A |
| A/duck/Liaoning/LN/2011 | H5N5 | 2.3.4 | EPI_ISL_290070 | 2011 | TPS | NNA | M | V | V | I | K | A |
| A/Environment/Hunan/18478/2014 | H5N1 | 2.3.4.4 | EPI_ISL_219776 | 2014 | MPS | NDA | M | V | V | T | T | T |
| A/duck/Eastern_China/S0808/2014 | H5N2 | 2.3.4.4 | EPI_ISL_208837 | 2014 | APS | NDA | M | V | V | I | R | A |
| A/chicken/Zhejiang/514135/2015 | H5N2 | 2.3.4.4 | EPI_ISL_279181 | 2015 | MPS | NDA | M | V | V | I | K | V |
| A/goose/Eastern_China/L1214/2012 | H5N8 | 2.3.4.4 | EPI_ISL_208830 | 2012 | MPS | NDA | M | V | I | I | K | A |
| A/duck/Guangdong/04.23_DGQTSJ124-P/2015(Mixed) | H5N2 | 2.3.4.4 | EPI_ISL_200540 | 2015 | PSF | DAY | M | V | V | I | K | A |

*H3 numbering
